# Theoretical laser cooling feasibility study of ZrH molecule at the fine structure level

**DOI:** 10.3389/fchem.2025.1603873

**Published:** 2025-07-25

**Authors:** Ghina Chamieh, Lokman Awad, Nayla El-Kork, Mahmoud Korek

**Affiliations:** ^1^Faculty of Science, Beirut Arab University, Beirut, Lebanon; ^2^Department of Physics, Khalifa University, Abu Dhabi, United Arab Emirates

**Keywords:** spectroscopic constants, spin-orbit coupling effect, laser cooling, franck-condon factors, radiative lifetime

## Abstract

A theoretical electronic structure calculation of the ZrH molecule is conducted via *ab initio* Complete Active Space Self-Consistent Field and the Multireference Configuration Interaction with Davidson correction calculation (CASSCF/MRCI + Q). The adiabatic potential energy curves (PECs) for the 53 low-lying electronic states in the representations of ^2s+1^Λ^(+/−)^ and Ω^(+/−)^ for ZrH molecule have been investigated along with the internuclear distance R_e_, the harmonic frequency ω_e_, the dipole moment μ, the rotational constant B_e_ and the electronic transition energy with respect to the ground state T_e_. are calculated. By using the canonical function approach, the vibrational energy E_v_, the rotational constants B_v_, the centrifugal constants D_v_, and the turning points R_min_ and R_max_ have been calculated up to the vibrational level v = 18. Based on the investigated data, the Franck−Condon factors, the Einstein coefficient, the radiative lifetimes, and the vibrational branching ratio for the transitions X^2^Δ_3/2_ - (1)^4^Φ_3/2_, X^2^Δ_3/2_ - (1)^4^Φ_5/2_, X^2^Δ_3/2_ - (1)^2^Π_3/2_ have been calculated. The large value of the radiative lifetimes in (ms) for these transitions proves that this molecule is not a good candidate for direct laser cooling.

## 1 Introduction

The characteristics of metal-hydrogen bonds and the function of metal d orbitals have led to an increase in the number of theoretical and experimental studies on transition metal hydrides. Both electron correlation and relativistic effects become significant for heavier transition metal hydrides. Generally speaking, low-lying electronic states with a variety of spatial and spin symmetries are closely clustered together in transition metal hydrides. This makes transition metal hydrides one of the most challenging possibilities for theoretical research, especially when combined with the electron correlation (McLean, 1983; Kraussand, 1985; Balasubraman et al., 1988; Balasubramanian et al., 1987; Hay and Martin, 1985).

Moreover, in the considered molecule, massive nuclear spins in the transition elements can produce complicated structural patterns with magnetic hyperfine structures (James et al., 1993). Chemistry may lead from the spectroscopy of these systems to better understand the bonding of transition metals, high-temperature chemical processes, and luminous chemical reactions (Langhoff and Bauschlicher, 1988; Korek and Hamdan, 2008). With a theoretical *ab initio* investigation, the electronic structures of diatomic molecules are required for astrophysics, astrochemistry, and laser cooling studies (Thompson and Ziurys, 2001).

The goal of the current study is to conduct a thorough theoretical analysis of several low-lying electronic states of the ZrH molecule, taking relativistic, electron correlation, and spin-orbit effects into account. SCF/SDCI/CPF calculations on two electronic states of ZrH have been performed by Langhoff et al. (1987a) without spin-orbit effects included. In this work, we perform a full active space (CASSCF/MRCI + Q) that incorporates the spin-orbit term on 36 electronic states of ZrH. To the best of our knowledge, there has been little theoretical and no experimental research done on the zirconium hydride ZrH molecule. This provided us with a strong incentive to examine the electronic structure of this molecule, as well as its spectroscopic characteristics and ro-vibrational studies. In the current work, the ab intio approach with an entire active space consistent field has been used to investigate the potential energy curves (PECs) of 53 electronic states for ZrH molecule in the ^2s+1^Λ^(+/−)^ and Ω^(+/−)^ representations. All these calculations are followed by a ro-vibrational analysis in order to determine the values of vibrational energy E_v_, the rotational constant B_v_, the centrifugal distortion constant D_v_, and the turning point abscissas R_min_ and R_max_. Based on these investigated data, the Franck−Condon factors, the Einstein coefficient, the radiative lifetimes, and the vibrational branching ratio are determined for the transitions X^2^Δ_3/2_ - (1)^4^Φ_3/2_, X^2^Δ_3/2_ - (1)^4^Φ_5/2_, X^2^Δ_3/2_ - (1)^2^Π_3/2._

## 2 Computational methods

The state average Complete Active Self Consistent Field (CASSCF)/Multireference Configuration Interaction (MRCI + Q) has been used to investigate the doublet and quartet electronic states of the ZrH molecule with and without the spin-orbit coupling. By using the Breit-Pauli operator and the ECP spin-orbit operator for the Zr-atom, the total Hamiltonian H_t_ = H_e_ + W_SO_ is diagonalized with the help of the Born-Oppenheimer approximation along with the spin-orbit perturbation. The lowest energies have been calculated for the spin-orbit coupling states Ω = 1/2, 3/2, and 5/2. With the graphical interface, GABEDIT (Werner et al., 2025), and the computational chemistry program MOLPRO (Allouche, 2011), these calculations have been accomplished. For the ZrH molecule, the ECP28MDF basis set (Peterson et al., 2007) is used for the Zr atom with 12 valence electrons distributed as *4s* ^*2*^ *4p* ^*6*^ *5s* ^*2*^ *4d* ^*2,*^ and the aug-cc-pV5Z basis set (Dunning, 1989) is considered for the H atom with one valence electron *1s* ^*1*^. Before we choose our basis set, we run several trials to choose the most accurate degeneracy between the states in the first and fourth symmetry. The ECP28MDF basis set and the aug-cc-pV5Z basis set gave the best degeneracy results.

As the MOPRO program can work only with the Abelian point group, the ZrH molecule is treated in C_2v_ instead of $C_{\infty }$ v. The active space for the considered ZrH molecule is *6σ (Zr: 4d*_*0,*_ *4d*_*2+*_ *, 5P*_*0*_ *, 5s*_*1*_ *; H:1s,2s), 2π (Zr: 4d*_*± 1,*_ *5p*_*± 1*_ *) and 1δ (Zr: 5d*_*2-*_ *),* where the corresponding irreducible representation is 6*a*_1_, 2*b*_*1*_, 2*b*_*2*_, and 1*a*_*2*_ noted by [6,2,2,1], In order to obtain the potential energy curves, the estimated energy points are connected using the avoided-crossing rule for electronic states that belong to the same irreducible representation of the single/double point group $C_{\infty V}$ . The one-dimensional Born-Oppenheimer Schrödinger equation is used to obtain the spectroscopic constants including R_e_ (equilibrium bond length), T_e_ (transition energy), ω_e_ (harmonic constant) and B_e_ (rotational constant). Due to the lack of experimental data on the ZrH molecule and its corresponding spectroscopic constants (R_e_, T_e_, ω_e,_ and B_e_), the comparison between our obtained results with any other experimental result was not possible.

## 3 Results and discussion

**FIGURE 1:**
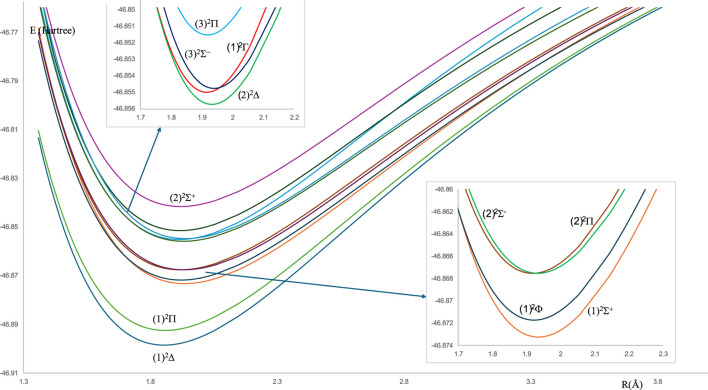
The spin-free potential energy curves of the doublet electronic states of the ZrH molecule.

**FIGURE 2:**
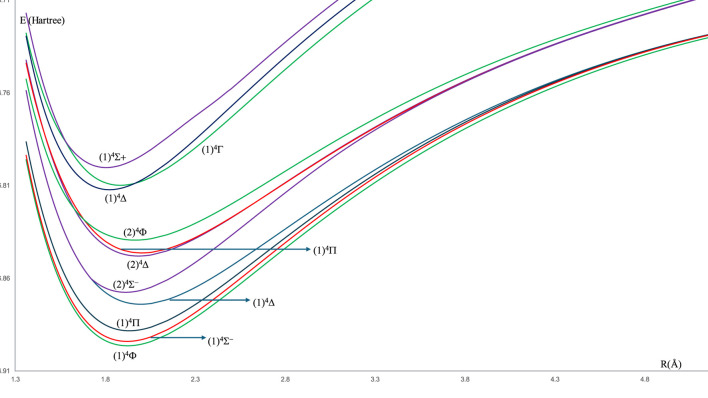
The spin-free potential energy curves of the quartet electronic states of the ZrH molecule.

**FIGURE 3:**
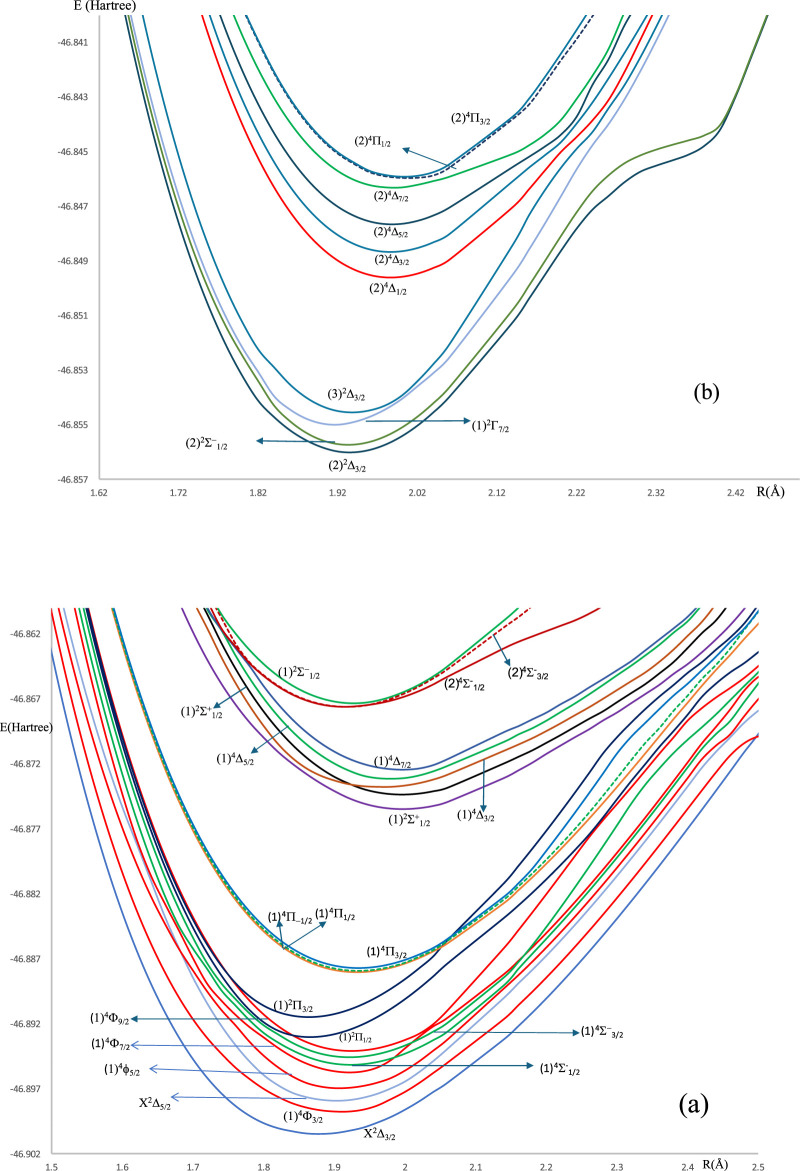
**(a)** The spin-orbit coupling potential energy curves in the range of −46.902 → −46.862 Hartree of ZrH molecule. **(b)** The spin-orbit coupling potential energy curves in the range of 46.857 → −46.841 Hartree of ZrH molecule.

For the spin-free ZrH molecule, the potential energy curves (PEC) using MRCI calculation for 22 doublet and quartet electronic states are investigated and plotted in Figures 1, 2 as a function of internuclear distance R in the ranges 1.20 Å ≤ R ≤ 2.20 Å and 1.20 Å ≤ R ≤ 4.80Å, respectively. For the spin-orbit coupling of the ZrH molecule, we investigated 31 electronic states where the corresponding potential energy curves are plotted in Figures 3a,b, where the ranges of energies are −46.902 → −46.862 Hartree and −46.857 → −46.841 Hartree, respectively. For the considered molecule ZrH, all of the studied states with spin-free and spin-orbit coupling are bound states, with depth potential energy curves indicating the strength of the bond and the stability of this molecule.

### 3.1 Spectroscopic parameters

For the studied ZrH molecule, the spectroscopic constants have been calculated by adapting a polynomial of R around the internuclear distance at equilibrium R_e_. These constants include the harmonic vibrational frequencies ω_e_, the relative energy with respect to the ground state T_e_, the internuclear distances R_e_, and the rotational constants B_e_. Tables 1, 2 provide these values for the various electronic states, along with those found in the literature for spin-orbital coupling and spin-free coupling. By comparing our outcomes of ω_e_ with those given in literature by Balasubramanian and Wang (1989), Langhoff et al. (1987b), we obtain a good accuracy with the relative differences Δω_e_/ω_e_ = 2.4%, 4.5%, 4.7%, and 6.8% for the electronic states X^2^Δ, (1)^4^Φ, (1)^4^Σ^−^ and (1)^2^Π respectively. While the comparison of our calculated values of R_e_ with those given in the literature (Balasubramanian and Wang, 1989) also shows a good agreement with the relative differences 3.0%, 5.6%, 5.3%, and 3.8% for the electronic states X^2^Δ, (1)^4^ Φ, (1)^4^Σ^−^ and (1)^2^Π respectively.

**TABLE 1:** The spin-free spectroscopic constants T_e_, R_e_, ω_e_, and B_e_ of the molecule ZrH.

State	T_e_ (cm^-1^)	ω_e_ (cm^-1^)	Δω_e_/ω_e_ %	R_e_ (Å)	ΔR_e_/R_e_ %	B_e_ (cm^-1^)
X^2^Δ	0.00^a^ 0.00^b^	1702.72^a^ 1743.00^b^ 1580^c^	2.40 7.21	1.8563^a^ 1.7990 ^b^ 1.8458^c^	3.08 0.56	4.9132 ^a^
(1)^4^Φ	448.52 ^a^	1536.17 ^a^ 1605.00 ^b^ 1525^c^	4.49 0.73	1.9250 ^a^ 1.8170 ^b^ 1.9013^c^	5.91 4.18	4.5655 ^a^
(1)^4^Σ^−^	972.32 ^a^	1540.69 ^a^ 1613.00 ^b^	4.74	1.9225 ^a^ 1.8190 ^b^	5.38	4.5730 ^a^
(1)^2^Π	1315.27 ^a^	1631.23 ^a^ 1742.00 ^b^	6.81	1.8599 ^a^ 1.7990 ^b^	3.27	4.8907 ^a^
(1)^4^Π	2220.38 ^a^	1512.54 ^a^ 1585.00 ^b^	4.83	1.9341 ^a^ 1.8590 ^b^	3.88	4.5220 ^a^
(1)^4^Δ	5362.11 ^a^	1423.74 ^a^ 1430.00 ^b^	0.49	1.9976 ^a^ 1.9440 ^b^	2.68	4.2346 ^a^
(1)^2^Σ^+^	5535.22 ^a^	1530.38 ^a^		1.9367 ^a^		4.5093 ^a^
(1)^2^ Φ	5873.74 ^a^	1520.14 ^a^		1.9237 ^a^		4.5716 ^a^
(2)^2^Π	6795.06 ^a^	1520.33 ^a^ 1564.00 ^b^	2.89	1.9196 ^a^ 1.8460 ^b^	3.83	4.5913 ^a^
(2)^2^Σ^−^	9381.77 ^a^	1490.55 ^a^		1.9331 ^a^		4.5230 ^a^
(1)^2^Γ	9543.48 ^a^	1569.80 ^a^		1.9162 ^a^		4.6075 ^a^
(4)^2^Δ	9594.28 ^a^	1497.07 ^a^		1.9400 ^a^		4.4890 ^a^
(3)^2^Π	10311.61 ^a^	1544.24 ^a^		1.9191 ^a^		4.5933 ^a^
(2)^4^Δ	11111.38 ^a^	1388.24 ^a^ 1478.00 ^b^	6.48	1.9854 ^a^ 1.9120 ^b^	3.70	4.2929 ^a^
2)^4^Π	11460.11 ^a^	1330.32 ^a^		2.0047 ^a^		4.2015 ^a^
(2)^2^Σ^+^	12511.39 ^a^	1506.85 ^a^ 1597.00 ^b^	6.04	1.9216 ^a^ 1.9040 ^b^	0.92	4.5805 ^a^
(2)^4^Φ	12996.86 ^a^	1256.94 ^a^		1.9666 ^a^		4.3586 ^a^
(3)^4^Δ	18971.02 ^a^	1747.22 ^a^		1.8239 ^a^		5.0853 ^a^
(1)^4^Γ	19491.38 ^a^	1625.85 ^a^		1.8848 ^a^		4.7627 ^a^
(1)^4^Σ^+^	21572.80 ^a^	1844.76 ^a^		1.8055 ^a^		5.2114 ^a^

^a^ Present work.

^b^ Ref. (Balasubramanian and Wang, 1989).

^c^ Ref. [20-SDCI, method].

**TABLE 2:** The spin-orbital coupling spectroscopic constants Te, Re, ωe, and Be of the molecule ZrH.

State	T_e_ (cm^-1^)	ω_e_ (cm^-1^)	Δ ω_e_/ω_e_ %	R_e_ (Å)	ΔR_e_/R_e_ %	B_e_ (cm^-1^)
X^2^Δ_3/2_	0.00^a^ 0.00^b^	1616.00 ^a^ 1777.00 ^b^	9.96	1.8779 ^a^ 1.770 ^b^	5.75	4.7958 ^a^
(1)^4^Φ_3/2_	371.97^a^	1621.02 ^a^ 1604.00 ^b^	1.05	1.9065 ^a^ 1.8200 ^b^	4.54	4.5697 ^a^
X^2^Δ_5/2_	555.05 ^a^	1566.95 ^a^ 1779.00 ^b^	13.6	1.9015 ^a^ 1.7700 ^b^	6.92	4.6950 ^a^
(1)^4^ϕ_5/2_	757.05 ^a^	1705.55 ^a^ 1604.00 ^b^	5.9	1.9113 ^a^ 1.8200 ^b^	9.59	4.6280 ^a^
(1)^4^Φ_7/2_	1022.76 ^a^	1895.83 ^a^ 1857.00 ^b^	6.75	1.9142 ^a^ 1.8200 ^b^	4.92	4.6292 ^a^
(1)^4^Σ^-^_1/2_	1161.52 ^a^	1547.54 ^a^ 1613.00 ^b^	4.27	1.9247 ^a^ 1.8200 ^b^	5.44	4.4970 ^a^
(1)^4^Σ^−^_3/2_	1305.00 ^a^	1503.53 ^a^ 1613.00 ^b^	7,32	1.9212 ^a^ 1.8200 ^b^	5.23	4.5892 ^a^
(1)^4^Φ_9/2_	1390.40 ^a^	1587.28 ^a^ 1606.00 ^b^	1.38	1.9289 ^a^ 1.8200 ^b^	5.65	4.5122 ^a^
(1)^2^Π_1/2_	1627.40 ^a^	1820.17 ^a^ 1740.00 ^b^	4.39	1.8678 ^a^ 1.7800 ^b^	4.70	4.8547 ^a^
(1)^2^Π_3/2_	1971.57 ^a^	1728.32 ^a^ 1740.00 ^b^	0.69	1.8639 ^a^ 1.7800 ^b^	4.50	4.8789 ^a^
(1)^4^Π_1/2_	2748.43 ^a^	1544.95 ^a^ 1585.00 ^b^	2.65	1.9340 ^a^ 1.8200 ^b^	5.90	4.5207 ^a^
(1)^4^Π_3/2_	2801.28 ^a^	1526.00 ^a^ 1585.00 ^b^	3.87	1.9345 ^a^ 1.8200 ^b^	5.92	4.5200 ^a^
(1)^4^Δ_1/2_	5481.13 ^a^	1538.04 ^a^ 1413.00 ^b^	8.12	1.9970 ^a^ 1.9200 ^b^	3.89	4.2332 ^a^
(1)^4^Δ_5/2_	5733.63 ^a^	1495.94 ^a^ 1413.00 ^b^	5.48	1.9954 ^a^ 1.9200 ^b^	3.78	4.2094 ^a^
(1)^2^Σ^+^_1/2_	5860.64 ^a^	1351.82 ^a^ 1598.00 ^b^	18.28	1.9609 ^a^ 1.8500 ^b^	5.66	4.4190 ^a^
(1)^4^Δ_3/2_	5998.14 ^a^	1663.94 ^a^ 1413.00 ^b^	15.03	1.9788 ^a^ 1.9200 ^b^	2.97	4.2871 ^a^
(1)^4^Δ_7/2_	6148.07 ^a^	1691.20 ^a^ 1414.00 ^b^	16.38	1.9908 ^a^ 1.9200 ^b^	3.56	4.2228 ^a^
(2)^4^Σ^−^_1/2_	7212.40 ^a^	1427.22 ^a^		1.9183 ^a^		4.6132 ^a^
(2)^4^Σ^−^_3/2_	7221.67 ^a^	1502.51 ^a^		1.9191 ^a^		4.4155 ^a^
(1)^2^Σ^−^_1/2_	7278.37 ^a^	1569.16 ^a^		1.9272 ^a^		4.5345 ^a^
(2)^2^Δ_3/2_	9753.50 ^a^	1592.08 ^a^ 1534.00 ^b^		1.9365 ^a^ 1.8800 ^b^		4.3692 ^a^
(2)^2^Σ^−^_1/2_	9809.65 ^a^	1546.22 ^a^		1.9336 ^a^		4.5280 ^a^
(1)^2^Γ_7/2_	9972.41 ^a^	1668.07 ^a^		1.9157 ^a^		4.6547 ^a^
(3)^2^Δ_3/2_	10074.06 ^a^	1561.81 ^a^		1.9374 ^a^		4.4756 ^a^
(2)^4^Δ_1/2_	11155.77 ^a^	1464.04 ^a^ 1518.00 ^b^	3.68	1.9865 ^a^ 1.8800 ^b^	5.36	4.3107 ^a^
(2)^4^Δ_3/2_	11363.45 ^a^	1420.14 ^a^ 1512.00 ^b^	6.48	1.9864 ^a^ 1.8800 ^b^	5.36	4.2780 ^a^
(2)^4^Δ_5/2_	11585.26 ^a^	1331.49 ^a^ 1511.00 ^b^	13.52	1.98 84 ^a^ 1.8800 ^b^	5.45	4.2037 ^a^
(2)^4^Δ_7/2_	11880.28 ^a^	1412.55 ^a^ 1513.00 ^b^	7.15	1.98 80 ^a^ 1.8800 ^b^	5.43	4.1870 ^a^
(2)^4^Π_1/2_	11951.13 ^a^	1667.16 ^a^		2.0040 ^a^		4.2120 ^a^
(2)^4^Π_3/2_	11969.82 ^a^	1733.68 ^a^ 1551.00 ^b^	10.51	2.0012 ^a^ 1.8800 ^b^	6.06	4.2379 ^a^

^a^ Present work.

^b^ Ref. (Balasubramanian and Wang, 1989).

Similarly, for the spin-orbital coupling, our calculated data strongly matched with what had been published in the literature for ω_e_ with relative differences Δω_e_/ω_e_ = 9.0%, 1.1%, 5.9% and 4.2% for the electronic states X^2^Δ_3/2_, (1)^4^Φ_3/2_, (1)^4^Φ_5/2_ and (1)^4^Σ^-^_1/2_ respectively. Moreover, the relative difference in the internuclear distances R_e_ for the electronic states X^2^Δ_3/2_, (1)^4^ Φ_3/2_, (1)^4^Φ_5/2_, and (1)^4^Σ^−^ also shows a very good agreement with relative differences of 4.6%, 4.4%, 4.7% and 5.2% for the states mentioned above respectively.

### 3.2 Ro-vibrational parameters

The rovibrational constants of the ZrH molecule, namely, the vibrational energy E_v_, the rotational constant B_v_, the centrifugal distortion constant D_v_, and the abscissas of the turning point R_min_ and R_max_ have been determined up to v = 18 for the spin-free and up to v = 14 for the spin-orbital coupling, respectively, using the canonical function approach (Korek and El-Kork, 2018; Zeid et al., 2018; Chmaisani et al., 2019) with the cubic spline interpolation. Tables 3 provide the electronic states (X)^2^Δ and (1)^2^Σ^+^ for the spin-free ZrH molecule, while Tables 4 provide the spin-orbital electronic states X^2^Δ_3/2_, X^2^Δ_3/2_, and (1)^2^Φ_3/2_. Additionally, 25 rovibrational spin-free electronic states have been studied with 26 spin-orbit coupling electronic states that are provided in Supplementary Tables TS1, TS2. The rovibrational constants of some electronic states are absent because of the crossing or avoided crossing of the corresponding potential energy curves. There is no comparison of these values with other results since they are calculated here for the first time.

**TABLE 3:** The spin-free rovibrational constants for the different vibrational levels of (X)^2^Δ and (1)^2^Σ^+^ electronic states of ZrH molecule.

(X)^2^Δ					
v	E_v_ (cm^-1^)	B_v_ (cm^-1^)	D_v_ × 10^4^ (cm^-1^)	R_min_ (Å)	R_max_ (Å)
0	833.54	4.891	1.71	1.730	2.011
1	2470.72	4.797	1.69	1.641	2.141
2	4070.38	4.705	1.68	1.591	2.240
3	5632.92	4.614	1.66	1.550	2.331
4	7159.28	9.867	1.76	1.521	2.410
5	8649.99	10.185	1.57	1.491	2.491
6	10104.37	10.147	4.21	1.460	2.561
7	11521.54	10.874	8.60	1.442	2.630
8	12902.26	10.852	2.12	1.422	2.700
9	14247.71	11.230	1.28	1.411	2.772
10	15558.44	11.204	2.80	1.390	2.851
11	16835.28	11.607	1.48	1.381	2.910

(1)^2^Σ^+^					
v	E_v_ (cm^-1^)	B_v_ (cm^-1^)	D_v_ × 10^4^ (cm^-1^)	R_min_ (Å)	R_max_ (Å)
0	761.27	4.493	1.59	1.798	2.098
1	2256.17	4.404	1.57	1.711	2.235
2	3716.35	4.317	1.55	1.656	2.340
3	5142.66	4.231	1.53	1.615	2.431
4	6535.33	9.299	0.76	1.581	2.516
5	7893.56	9.275	2.28	1.553	2.598
6	9217.30	9.568	1.89	1.528	2.676
7	10507.83	10.211	0.38	1.507	2.752
8	11765.86	9.847	3.38	1.487	2.828
9	12992.03	10.178	2.20	1.470	2.902
10	14187.09	10.522	1.29	1.454	2.975
11	15351.63	10.499	2.70	1.439	3.049
12	16486.34	10.865	1.42	1.425	3.122
13	17591.79	10.843	2.75	1.413	3.195
14	18668.58	11.230	1.28	1.401	3.268

**TABLE 4:** The spin-orbit coupling rovibrational constants for the different vibrational levels of (X^2^)Δ_3/2_, (1)^2^Φ_3/2_, and X^2^Δ_5/2_ electronic states of ZrH molecule.

X^2^Δ_3/2_					
v	E_v_ (cm^-1^)	B_v_ (cm^-1^)	D_v_ × 10^4^ (cm^-1^)	R_min_ (Å)	R_max_ (Å)
0	760.42	4.765	2.03	1.736	2.042
1	2162.78	4.631	1.85	1.655	2.189
2	3580.18	8.972	3.29	1.604	2.296
3	4987.26	9.242	3.49	1.565	2.389
4	6350.01	9.859	1.04	1.534	2.474

(1)^2^Φ_3/2_					
v	E_v_ (cm^-1^)	B_v_ (cm^-1^)	D_v_ × 10^4^ (cm^-1^)	R_min_ (Å)	R_max_ (Å)
0	767.84	4.688	1.85	1.758	2.052
1	2247.41	4.604	2.01	1.665	2.198
2	3655.14	9.116	7.80	1.612	2.303
3	5049.61	9.391	8.44	1.571	2.395
4	6296.31	9.676	24.5	1.542	2.489

X^2^Δ_5/2_					
v	E_v_ (cm^-1^)	B_v_ (cm^-1^)	D_v_ × 10^4^ (cm^-1^)	R_min_ (Å)	R_max_ (Å)
0	815.91	4.659	1.54	1.769	2.054
1	2412.54	4.542	1.71	1.686	2.196
2	3910.72	4.411	1.79	1.634	2.307
3	5339.73	9.450	17.7	1.588	2.401
4	6686.59	10.106	3.05	1.553	2.510
5	7932.11	10.058	17.5	1.525	2.602
6	9151.24	10.402	11.9	1.501	2.674
7	10401.30	10.359	18.6	1.478	2.748

### 3.3 Laser cooling and electronic transition dipole moment

The slight difference in the internuclear distance at equilibrium positions (Table 2) between the ground X^2^Δ_3/2_ and the seven excited (1)^4^Φ_3/2_, (1)^4^Φ_5/2_, (1)^2^Π_1/2_, (1)^2^Π_3/2_, (1)^4^Π_-1/2_, (1)^4^Π_1/2_, and (1)^4^Π_3/2_ states incited us to study the suitability of the molecule ZrH for laser cooling for the transition between the ground and these seven states. The transitions between the ground and the other low-lying excited states in Figure 3a are forbidden. The transition X^2^Δ_3/2_ - (1)^2^Π_1/2_ can not be considered for laser cooling because of the intersection of the PEC of state (1)^2^Π_1/2_ with that of (1)^4^Φ_9/2_ state at 22 cm^-1^ from the ground, which can perturb the cooling cycling between these two states. Similarly, and because of the same reason, there is no cooling between the ground X^2^Δ_3/2_ and the states (1)^4^Π_-1/2_, (1)^4^Π_1/2_, and (1)^4^Π_3/2_ because of the intersections of their PEC with that of (1)^2^Π_3/2_.

**FIGURE 4:**
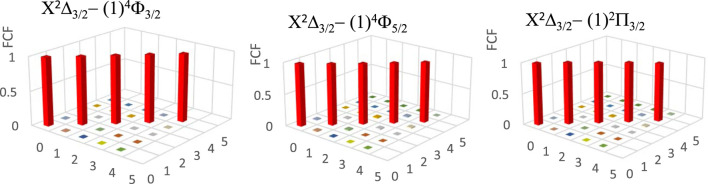
Franck-Condon factor for the transitions X^2^Δ_3/2_ - (1)^4^Φ_3/2_, X^2^Δ_3/2_ - (1)^4^Φ_5/2_, X^2^Δ_3/2_ - (1)^2^Π_3/2_ of the ZrH molecule.

The three main conditions for laser cooling for a molecule are a diagonal Franck-Condon factor (FCF), a short radiative lifetime, and the absence of an intermediate state disturbing the cycling process between the two studied electronic states. Figure 4 shows the diagonality of the calculated FCF for the transitions X^2^Δ_3/2_ - (1)^4^Φ_3/2_, X^2^Δ_3/2_ - (1)^4^Φ_5/2_, X^2^Δ_3/2_ - (1)^2^Π_3/2_ of the ZrH molecule by using the LEVEL 11 program (Le Roy, 2017). Having the diagonality of the FCF of this transition, we have to find the vibrational branching ratio loss R_v’v_ for these transitions between the two vibrational levels v' and v, which is given by (Equation 1)

$$R_{\nu ’v}=\frac{A_{\nu^{\prime }\nu }\;}{\sum_{\nu }A_{\nu^{\prime }\nu }}$$ (1)

where $A_{\nu^{\prime }\nu }$ is the Einstein coefficients (Equation 2)

$$A_{\nu^{\prime }\nu }=\left(\left(3.1361891\right)\left(10^{-7}\right){\left(\Delta E\right)}^{3}{\left(\langle \left.\psi_{\nu^{\prime }}\right|M\left(r\right)\left|\psi_{\nu }\right.\rangle \right)}^{2}\right)$$ (2)

**FIGURE 5:**
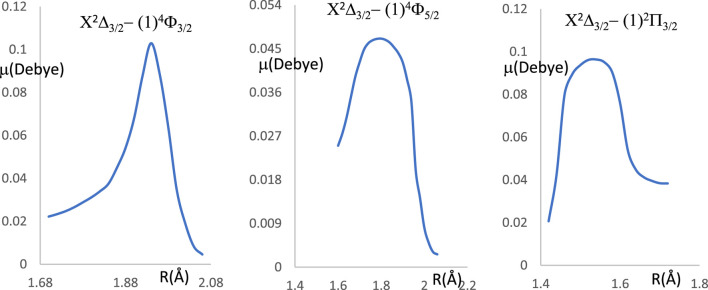
The transition dipole moment curves for the transitions X^2^Δ_3/2_ - (1)^4^Φ_3/2_, X^2^Δ_3/2_ - (1)^4^Φ_5/2_, X^2^Δ_3/2_ - (1)^2^Π_3/2_ of the ZrH molecule.

Between the two studied vibrational levels v and v', ΔE is the energy difference, and Μ(r) is the electronic transition dipole moment between the two electronic states that are considered (in Debye). By using the quantum chemistry program MOLPRO (Allouche, 2011), this transition dipole moment is calculated and plotted in Figure 5. The transition strength in electronic and other types of spectroscopy depends on the symmetry and orbital contributions. Generally, weak transitions occur between the same symmetries of the transitions, while strong transitions are obtained between different symmetries. From this Figure, one can notice that the transition dipole moment is larger for the higher spin than that of the lower one. The calculated values of the branching ratio loss R_v’v_ and the Einstein coefficients for the studied vibrational levels are given in Table 5 for the three transitions X^2^Δ_3/2_ - (1)^4^Φ_3/2_, X^2^Δ_3/2_ − (1)^4^Φ_5/2_, X^2^Δ_3/2_ - (1)^2^Π_3/2_. Based on these calculated data, the investigated values of the radiative lifetime, which is given by τ (s) = 1/A_ν’ν_ for these transitions of the molecule ZrH, are given in Table 5. These large values of the radiative lifetime 0.094 ms˂τ˂13.651 ms show the non-availability of the molecule ZrH for laser cooling for these three transitions.

**TABLE 5:** The radiative lifetimes τ, and the vibrational branching ratio of the vibrational transitions X^2^Δ_3/2_ - (1)^4^Φ_3/2_, X^2^Δ_3/2_ - (1)^4^Φ_5/2_, X^2^Δ_3/2_ - (1)^2^Π_3/2_ of the molecule ZrH.

X^2^Δ_3/2_ - (1)^4^Φ_3/2_						
	ν′ (1)^4^Φ_3/2_) = 0		1	2	3	4
ν (X^2^Δ_3/2_) = 0	Aν ν′	0.165253973	0.9953492	2.029674292	134.16564	44.420156
	Rν ν′	1.21E-03	2.26E-03	2.70E-03	9.97E-02	1.31E-02
ν = 1	Aν ν′	0.546325036	0.8298343	63.2809528	439.56342	340.10572
	Rν ν′	4.01E-03	1.89E-03	8.43E-02	3.27E-01	1.00E-01
ν = 2	Aν ν′	0.564105915	10.55921	6.504155551	511.71883	1288.8692
	Rν ν′	4.15E-03	2.40E-02	8.66E-03	3.80E-01	3.79E-01
ν = 3	Aν ν′	80.89510655	188.65456	82.24372971	24.795765	1631.9996
	Rν ν′	5.94E-01	4.29E-01	1.10E-01	1.84E-02	4.80E-01
ν = 4	Aν ν′	53.90876952	238.42653	596.7719993	235.59598	96.158914
	Rν ν′	3.96E-01	5.43E-01	7.95E-01	1.75E-01	2.83E-02
Sum (s^-1^) = A_ν’ν_		136.079561	439.46548	750.8305116	1345.8396	3401.5536
τ:(s) = 1/A_ν’ν_		0.007348642	0.0022755	0.001331859	0.000743	0.000294
τ:(s) = ms_ν_		7.348642	2.2755	1.331859	0.743	0.294

Transition X^2^Δ_3/2_ – (1)^4^Φ_5/2_						
	ν′ (1)^4^Φ_5/2_) = 0		1	2	3	4
ν (X^2^Δ_3/2_) = 0	Aν ν′	619.0361779	3.322961	1.597246154	0.0523209	6.662E-13
	Rν ν′	1.00E+00	1.70E-03	7.53E-04	2.46E-05	3.03E-16
ν = 1	Aν ν′	−0.053145577	1956.5203	5.347791897	1.5400587	0.0994297
	Rν ν′	−8.59E-05	9.98E-01	2.52E-03	7.25E-04	4.53E-05
ν = 2	Aν ν′	−0.088394011	−0.03883	2114.03632	6.8553459	1.3061967
	Rν ν′	−1.43E-04	−1.98E-05	9.97E-01	3.23E-03	5.95E-04
ν = 3	Aν ν′	−0.000119448	−0.059971	−0.034182559	2116.5003	6.4603686
	Rν ν′	−1.93E-07	−3.06E-05	−1.61E-05	9.96E-01	2.94E-03
ν = 4	Aν ν′	−0.000622017	−0.000438	−0.039899125	−0.017109	2187.7551
	Rν ν′	−1.01E-06	−2.23E-07	−1.88E-05	−8.05E-06	9.96E-01
Sum (s^-1^) = A_ν’ν_		618.8938969	1959.744	2120.907277	2124.9309	2195.6211
τ:(s) = 1/A_ν’ν_ τ:(s) = ms_ν_		0.001615786 1.615786	0.0005103 0.5103	0.000471496 0.471496	0.0004706 0.4706	0.0004555 0.4555

Transition X^2^Δ_3/2_ - (1)^2^Π_3/2_						
	ν′ ((1)^2^Π_3/2_) = 0		1	2	3	4
ν (X^2^Δ_3/2_) = 0	A_νν'_	62.68084973	62.68085	106.064685	38.411363	0.0418764
	R_νν'_	8.56E-01	8.56E-01	2.15E-02	4.95E-03	3.95E-06
ν = 1	A_νν'_	7.189385455	7.1893855	2976.533882	84.124641	145.06092
	R_νν'_	9.81E-02	9.81E-01	6.04E-01	1.08E-02	1.37E-02
ν = 2	A_νν'_	0.186822923	0.1868229	1766.318525	4456.5469	0.01118
	R_νν'_	2.55E-03	2.55E-03	3.58E-01	5.74E-01	1.05E-06
ν = 3	A_νν'_	2.623026497	2.6230265	81.11997274	3014.4732	5478.05
	R_νν'_	3.58E-02	3.58E-02	1.65E-02	3.88E-01	5.16E-01
ν = 4	A_νν'_	0.572876671	0.5728767	0.137005879	166.26142	4991.626
	R_νν'_	7.82E-03	7.82E-03	2.78E-05	2.14E-02	4.70E-01
Sum (s^-1^) = A_ν’ν_		73.25296128	73.252961	4930.17407	7759.8175	10614.79
τ:(s) =/A_ν’ν_		0.013651325	0.0136513	0.000202833	0.0001289	9.421E-05
τ: (ms)		13.65132525	13.651325	0.202832595	0.128869	0.0942082

## 4 Conclusion

In the current work, an *ab initio* calculation using the Complete Active Space Self-Consistent Field/Multireference Configuration Interaction with Davidson corrective calculation (CASSCF/MRCI + Q) was carried out for doublet and quartet 53 low-lying electronic states of the ZrH molecule with and without spin-orbit coupling effect. The comparison of our calculated values of the spectroscopic constants R_e_ and ω_e_ with those available in the literature shows good accuracy with the average relative differences Δω_e_/ω_e_ = 4.6% and ΔR_e_/R_e_ = 5.05% for the free spin calculation and Δω_e_/ω_e_ = 4.42% and ΔR_e_/R_e_ = 4.72% for the spin-orbit coupling calculation for the states X^2^Δ, (1)^4^Φ, (1)^4^Σ^−^ and (1)^2^Π. By using the canonical function approach, the rovibrational calculation of the constants E_v_, B_v_, D_v_, R_min_, and R_max_ has been performed; there is no comparison of these values with other results since they are calculated here for the first time. The calculation of the Franck−Condon factors, the Einstein coefficients, the vibrational branching ratios, and the large values of the radiative lifetimes in (ms) for the transitions between the ground and the low-lying permitted transitions shows the non-availability of the molecule ZrH for direct laser cooling.

## Data Availability

The original contributions presented in the study are included in the article/Supplementary Material, further inquiries can be directed to the corresponding author.
