# Supplementary material for: Theoretical laser cooling feasibility study of ZrH molecule at the fine structure level
Source: Front Chem. 2025 Jul 25;13:1603873. doi: 10.3389/fchem.2025.1603873 (PMC12331741; doi:10.3389/fchem.2025.1603873)
Supplement: Supplementary file 1 [file Table1.docx]

Table (TS1): The rovibrational constants for the different vibrational levels of different electronic states of the spin-free ZrH molecule.

| (2)^2^Δ | | | | | |
| --- | --- | --- | --- | --- | --- |
| v | E_v_  (cm^-1^) | B_v_  (cm^-1^) | D_v_ ×10^4^  (cm^-1^) | R_min_  (Å) | R_max_  (Å) |
| 0 | 739.61 | 4.506 | 1.70 | 1.793 | 2.097 |
| 1 | 2189.45 | 4.411 | 1.68 | 1.705 | 2.237 |
| 2 | 3602.09 | 4.316 | 1.67 | 1.650 | 2.345 |
| 3 | 4977.90 | 4.223 | 1.65 | 1.609 | 2.440 |
| 4 | 6317.74 | 9.644 | 0.59 | 1.576 | 2.528 |
| 5 | 7622.22 | 9.298 | 5.75 | 1.547 | 2.616 |
| 6 | 8892.04 | 9.596 | 4.84 | 1.523 | 2.694 |
| 7 | 10127.76 | 10.259 | 1.02 | 1.501 | 2.774 |
| 8 | 11330.00 | 10.240 | 2.50 | 1.482 | 2.852 |
| 9 | 12499.47 | 10.585 | 1.55 | 1.465 | 2.930 |
| 10 | 13636.84 | 10.564 | 3.39 | 1.450 | 3.007 |
| 11 | 14742.79 | 10.931 | 1.84 | 1.435 | 3.084 |
| 12 | 15818.07 | 10.910 | 3.74 | 1.421 | 3.161 |
| 13 | 16863.24 | 11.298 | 1.81 | 1.409 | 3.239 |
| 14 | 17879.06 | 11.280 | 3.43 | 1.398 | 3.332 |

| (1)^2^Γ | | | | | | |
| --- | --- | --- | --- | --- | --- | --- |
| v | E_v_  (cm^-1^) | B_v_  (cm^-1^) | D_v_ ×10^4^  (cm^-1^) | R_min_  (Å) | R_max_  (Å) |  |
| 0 | 779.38 | 4.589 | 1.61 | 1.780 | 2.076 |  |
| 1 | 2310.10 | 4.500 | 1.60 | 1.693 | 2.211 |  |
| 2 | 3805.62 | 4.411 | 1.57 | 1.639 | 2.314 |  |
| 3 | 5267.02 | 4.324 | 1.56 | 1.599 | 2.405 |  |
| 4 | 6694.41 | 9.289 | 1.71 | 1.565 | 2.488 |  |
| 5 | 8086.77 | 9.581 | 1.55 | 1.537 | 2.568 |  |
| 6 | 9443.85 | 9.547 | 4.15 | 1.512 | 2.646 |  |
| 7 | 10766.77 | 10.213 | 0.83 | 1.491 | 2.721 |  |
| 8 | 12056.34 | 10.194 | 2.03 | 1.472 | 2.795 |  |
| 9 | 13312.76 | 10.536 | 1.21 | 1.454 | 2.867 |  |
| 10 | 14536.89 | 10.514 | 2.63 | 1.438 | 2.942 |  |
| 11 | 15729.12 | 10.879 | 1.39 | 1.424 | 3.014 |  |
| 12 | 16889.98 | 10.857 | 2.77 | 1.410 | 3.087 |  |
| 13 | 18019.89 | 11.242 | 1.30 | 1.398 | 3.160 |  |
| 14 | 19119.47 | 11.223 | 2.41 | 1.386 | 3.233 |  |
| 15 | | 20189.30 | 11.193 | 4.23 | 1.376 | 3.307 |
| 16 | 21230.01 | 11.612 | 1.76 | 1.365 | 3.381 |  |

| (2)^2^Σ^+^ | | | | | |
| --- | --- | --- | --- | --- | --- |
| v | E_v_  (cm^-1^) | B_v_  (cm^-1^) | D_v_ ×10^4^  (cm^-1^) | R_min_  (Å) | R_max_  (Å) |
| 0 | 748.30 | 4.560 | 1.72 | 1.783 | 2.085 |
| 1 | 2215.36 | 4.464 | 1.71 | 1.695 | 2.224 |
| 2 | 3644.50 | 4.369 | 1.69 | 1.640 | 2.331 |
| 3 | 5036.92 | 4.275 | 1.68 | 1.600 | 2.425 |
| 4 | 6392.55 | 9.798 | 3.55 | 1.566 | 2.513 |
| 5 | 7712.19 | 9.760 | 0.11 | 1.538 | 2.597 |
| 6 | 8996.38 | 10.084 | 8.68 | 1.513 | 2.678 |
| 7 | 10245.91 | 10.794 | 1.57 | 1.492 | 2.757 |
| 8 | 11461.58 | 10.381 | 0.15 | 1.473 | 2.835 |
| 9 | 12643.74 | 10.750 | 9.54 | 1.456 | 2.912 |
| 10 | 13793.29 | 10.700 | 0.21 | 1.440 | 2.989 |
| 11 | 14910.57 | 11.101 | 0.12 | 1.425 | 3.066 |
| 12 | 15996.78 | 11.051 | 0.23 | 1.412 | 3.146 |
| 13 | 17052.60 | 11.479 | 0.20 | 1.400 | 3.219 |
| 14 | 18078.84 | 11.437 | 0.22 | 1.388 | 3.297 |

| (1)^2^Π | | | | | |
| --- | --- | --- | --- | --- | --- |
| v | E_v_  (cm^-1^) | B_v_  (cm^-1^) | D_v_ ×10^4^  (cm^-1^) | R_min_  (Å) | R_max_  (Å) |
| 0 | 809.73 | 4.872 | 1.79 | 1.726 | 2.016 |
| 1 | 2399.74 | 4.777 | 1.77 | 1.640 | 2.149 |
| 2 | 3952.61 | 4.683 | 1.75 | 1.587 | 2.250 |
| 3 | 5469.11 | 9.508 | 1.82 | 1.547 | 2.339 |
| 4 | 6950.44 | 10.143 | 0.54 | 1.515 | 2.421 |
| 5 | 8397.10 | 10.121 | 1.61 | 1.487 | 2.500 |
| 6 | 9808.33 | 10.454 | 1.27 | 1.463 | 2.575 |
| 7 | 11183.69 | 11.186 | 0.24 | 1.442 | 2.649 |
| 8 | 12524.20 | 10.781 | 2.14 | 1.423 | 2.722 |
| 9 | 13830.97 | 11.156 | 1.31 | 1.406 | 2.794 |
| 10 | 15104.63 | 11.127 | 2.75 | 1.391 | 2.865 |
| 11 | 16345.93 | 11.529 | 1.46 | 1.376 | 2.936 |
| 12 | 17555.51 | 11.945 | 0.70 | 1.363 | 3.006 |
| 13 | 18734.10 | 11.927 | 1.31 | 1.351 | 3.077 |
| 14 | 19882.32 | 11.900 | 2.29 | 1.340 | 3.148 |
| 15 | 21000.92 | 12.352 | 0.94 | 1.329 | 3.219 |
| 16 | 22090.54 | 12.330 | 1.50 | 1.319 | 3.290 |
| 17 | 23151.77 | 12.802 | 0.53 | 1.310 | 3.362 |
| 18 | 24185.10 | 12.786 | 0.70 | 1.301 | 3.435 |

| (1)^2^ Φ | | | | | |
| --- | --- | --- | --- | --- | --- |
| v | E_v_  (cm^-1^) | B_v_  (cm^-1^) | D_v_ ×10^4^  (cm^-1^) | R_min_  (Å) | R_max_  (Å) |
| 0 | 754.15 | 4.534 | 1.66 | 1.785 | 2.086 |
| 1 | 2233.81 | 4.442 | 1.65 | 1.697 | 2.224 |
| 2 | 3677.19 | 4.350 | 1.63 | 1.643 | 2.330 |
| 3 | 5085.28 | 4.260 | 1.61 | 1.602 | 2.423 |
| 4 | 6458.62 | 9.488 | .043 | 1.568 | 2.510 |
| 5 | 7797.73 | 9.471 | 1.28 | 1.540 | 2.592 |
| 6 | 9103.13 | 9.439 | 3.36 | 1.516 | 2.671 |
| 7 | 10375.37 | 10.087 | .073 | 1.494 | 2.748 |
| 8 | 11615.24 | 10.067 | 1.75 | 1.475 | 2.825 |
| 9 | 12823.29 | 10.403 | 1.09 | 1.457 | 2.900 |
| 10 | 14000.16 | 10.380 | 2.31 | 1.441 | 2.974 |
| 11 | 15146.49 | 10.739 | 1.26 | 1.427 | 3.049 |
| 12 | 16262.76 | 10.715 | 2.45 | 1.413 | 3.123 |
| 13 | 17349.53 | 11.096 | 1.18 | 1.401 | 3.198 |
| 14 | 18407.43 | 11.074 | 2.12 | 1.390 | 3.272 |
| 15 | 19437.11 | 11.475 | 0.90 | 1.379 | 3.347 |
| 16 | 20438.99 | 11.458 | 1.51 | 1.369 | 3.423 |

| (2)^2^Π | | | | | |
| --- | --- | --- | --- | --- | --- |
| v | E_v_  (cm^-1^) | B_v_  (cm^-1^) | D_v_ ×10^4^  (cm^-1^) | R_min_  (Å) | R_max_  (Å) |
| 0 | 754.27 | 4.570 | 1.70 | 1.781 | 2.082 |
| 1 | 2233.55 | 4.475 | 1.68 | 1.693 | 2.221 |
| 2 | 3676.21 | 4.380 | 1.66 | 1.639 | 2.327 |
| 3 | 5083.03 | 4.287 | 1.64 | 1.598 | 2.420 |
| 4 | 6454.57 | 9.545 | 0.52 | 1.565 | 2.507 |
| 5 | 7791.42 | 9.525 | 1.55 | 1.537 | 2.590 |
| 6 | 9094.09 | 9.829 | 1.24 | 1.513 | 2.670 |
| 7 | 10363.09 | 10.494 | 0.23 | 1.491 | 2.747 |
| 8 | 11599.31 | 10.127 | 2.09 | 1.472 | 2.824 |
| 9 | 12803.28 | 10.468 | 1.30 | 1.455 | 2.900 |
| 10 | 13975.63 | 10.443 | 2.74 | 1.439 | 2.975 |
| 11 | 15117.03 | 10.807 | 1.49 | 1.425 | 3.050 |
| 12 | 16228.07 | 10.781 | 2.89 | 1.412 | 3.125 |
| 13 | 17309.28 | 11.168 | 1.39 | 1.400 | 3.200 |
| 14 | 18361.30 | 11.144 | 2.49 | 1.388 | 3.275 |
| 15 | 19384.72 | 11.551 | 1.06 | 1.378 | 3.351 |
| 16 | 20380.08 | 11.533 | 1.78 | 1.368 | 3.427 |

| (3)^2^Π | | | | | |
| --- | --- | --- | --- | --- | --- |
| v | E_v_  (cm^-1^) | B_v_  (cm^-1^) | D_v_ ×10^4^  (cm^-1^) | R_min_  (Å) | R_max_  (Å) |
| 0 | 765.58 | 4.573 | 1.66 | 1.781 | 2.080 |
| 1 | 2265.92 | 4.478 | 1.65 | 1.694 | 2.218 |
| 2 | 3726.89 | 4.383 | 1.64 | 1.640 | 2.324 |
| 3 | 5148.84 | 4.289 | 1.63 | 1.600 | 2.417 |
| 4 | 6532.31 | 9.557 | 0.43 | 1.566 | 2.504 |
| 5 | 7877.53 | 9.540 | 1.27 | 1.539 | 2.587 |
| 6 | 9185.02 | 9.510 | 3.35 | 1.514 | 2.668 |
| 7 | 10455.20 | 10.162 | 0.70 | 1.493 | 2.747 |
| 8 | 11689.20 | 10.144 | 1.67 | 1.474 | 2.826 |
| 9 | 12887.59 | 10.482 | 1.02 | 1.457 | 2.903 |
| 10 | 14051.16 | 10.462 | 2.15 | 1.441 | 2.981 |
| 11 | 15180.87 | 10.822 | 1.15 | 1.427 | 3.058 |
| 12 | 16277.36 | 10.802 | 2.23 | 1.417 | 3.136 |
| 13 | 17341.42 | 11.183 | 1.06 | 1.404 | 3.214 |
| 14 | 18373.92 | 11.165 | 1.90 | 1.394 | 3.298 |

| (3)^2^Δ | | | | | |
| --- | --- | --- | --- | --- | --- |
| v | E_v_  (cm^-1^) | B_v_  (cm^-1^) | D_v_ ×10^4^  (cm^-1^) | R_min_  (Å) | R_max_  (Å) |
| 0 | 833.53 | 4.891 | 1.71 | 1.724 | 2.010 |
| 1 | 2470.71 | 4.798 | 1.69 | 1.640 | 2.141 |
| 2 | 4070.38 | 4.705 | 1.68 | 1.587 | 2.240 |
| 3 | 5632.94 | 4.614 | 1.66 | 1.548 | 2.328 |
| 4 | 7159.31 | 9.867 | 1.76 | 1.516 | 2.409 |
| 5 | 8650.03 | 10.185 | 1.57 | 1.488 | 2.485 |
| 6 | 10104.43 | 10.147 | 4.22 | 1.464 | 2.560 |
| 7 | 11521.64 | 10.875 | 0.86 | 1.443 | 2.632 |
| 8 | 12902.37 | 10.852 | 2.12 | 1.426 | 2.704 |
| 9 | 14247.80 | 11.230 | 1.29 | 1.407 | 2.775 |
| 10 | 15558.49 | 11.204 | 2.81 | 1.392 | 2.845 |
| 11 | 16835.31 | 11.608 | 1.49 | 1.378 | 2.915 |
| 12 | 18078.91 | 11.581 | 2.99 | 1.364 | 2.985 |
| 13 | 19289.97 | 12.010 | 1.40 | 1.352 | 3.055 |
| 14 | 20469.22 | 11.986 | 2.59 | 1.341 | 3.125 |
| 15 | 21617.38 | 11.949 | 4.54 | 1.331 | 3.195 |
| 16 | 22735.01 | 12.419 | 1.87 | 1.321 | 3.265 |
| 17 | 23822.75 | 12.391 | 3.10 | 1.312 | 3.337 |
| 18 | 24881.18 | 12.348 | 4.88 | 1.303 | 3 |

| (1)^2^Σ^−^ | | | | | |
| --- | --- | --- | --- | --- | --- |
| v | E_v_  (cm^-1^) | B_v_  (cm^-1^) | D_v_ ×10^4^  (cm^-1^) | R_min_  (Å) | R_max_  (Å) |
| 0 | 740.96 | 4.506 | 1.69 | 1.794 | 2.098 |
| 1 | 2196.81 | 4.420 | 1.67 | 1.705 | 2.236 |
| 2 | 3619.44 | 4.334 | 1.66 | 1.649 | 2.341 |
| 3 | 5009.30 | 4.249 | 1.64 | 1.607 | 2.434 |
| 4 | 6366.64 | 9.311 | 2.12 | 1.573 | 2.520 |
| 5 | 7691.39 | 9.602 | 1.98 | 1.544 | 2.602 |
| 6 | 8983.72 | 9.565 | 5.33 | 1.519 | 2.681 |
| 7 | c10244.17 | 10.231 | 1.19 | 1.497 | 2.758 |
| 8 | 11473.20 | 10.209 | 2.95 | 1.477 | 2.833 |
| 9 | 12671.29 | 10.554 | 1.89 | 1.459 | 2.909 |
| 10 | 13838.90 | 10.527 | 4.13 | 1.443 | 2.983 |
| 11 | 14976.46 | 10.896 | 2.33 | 1.428 | 3.057 |
| 12 | 16084.43 | 11.277 | 1.20 | 1.415 | 3.131 |
| 13 | 17163.19 | 11.261 | 2.38 | 1.402 | 3.206 |
| 14 | 18213.23 | 11.235 | 4.48 | 1.391 | 3.281 |
| 15 | 19234.98 | 11.649 | 2.02 | 1.380 | 3.356 |

| (2)^2^Σ^−^ | | | | | |
| --- | --- | --- | --- | --- | --- |
| v | E_v_  (cm^-1^) | B_v_  (cm^-1^) | D_v_ ×10^4^  (cm^-1^) | R_min_  (Å) | R_max_  (Å) |
| 0 | 740 | 4.506 | 1.70 | 1.793 | 2.097 |
| 1 | 2189 | 4.411 | 1.68 | 1.705 | 2.280 |
| 2 | 3602 | 4.316 | 1.67 | 1.650 | 2.345 |
| 3 | 4978 | 4.223 | 1.65 | 1.609 | 2.440 |
| 4 | 6318 | 9.643 | 0.58 | 1.576 | 2.529 |
| 5 | 7622 | 9.297 | 5.70 | 1.547 | 2.613 |
| 6 | 8892 | 9.595 | 4.79 | 1.523 | 2.694 |
| 7 | 10128 | 10.257 | 1.01 | 1.501 | 2.774 |
| 8 | 11330 | 10.239 | 2.47 | 1.482 | 2.852 |
| 9 | 12499 | 10.583 | 1.53 | 1.465 | 2.930 |
| 10 | 13637 | 10.563 | 3.36 | 1.450 | 3.007 |
| 11 | 14743 | 10.929 | 1.82 | 1.435 | 3.084 |
| 12 | 15818 | 10.908 | 3.70 | 1.421 | 3.161 |
| 13 | 16863 | 11.296 | 1.79 | 1.409 | 3.239 |
| 14 | 17879 | 11.278 | 3.40 | 1.398 | 3.316 |

| (4)^2^Δ | | | | | |
| --- | --- | --- | --- | --- | --- |
| v | E_v_  (cm^-1^) | B_v_  (cm^-1^) | D_v_ ×10^4^  (cm^-1^) | R_min_  (Å) | R_max_  (Å) |
| 0 | 749.07 | 4.472 | 1.62 | 1.801 | 2.103 |
| 1 | 2216.17 | 4.374 | 1.61 | 1.714 | 2.243 |
| 2 | 3643.78 | 4.277 | 1.59 | 1.660 | 2.351 |
| 3 | 5032.94 | 4.183 | 1.58 | 1.619 | 2.446 |
| 4 | 6384.56 | 9.255 | 0.53 | 1.586 | 2.535 |
| 5 | 7699.45 | 9.236 | 1.56 | 1.558 | 2.620 |
| 6 | 8978.70 | 9.525 | 1.28 | 1.534 | 2.701 |
| 7 | 10223.17 | 9.829 | 0.94 | 1.513 | 2.780 |
| 8 | 11433.80 | 9.806 | 2.23 | 1.493 | 2.858 |
| 9 | 12611.49 | 10.481 | 0.39 | 1.476 | 2.936 |
| 10 | 13757.07 | 10.103 | 3.05 | 1.460 | 3.013 |
| 11 | 14871.40 | 10.821 | 0.46 | 1.446 | 3.089 |
| 12 | 15955.29 | 10.421 | 3.38 | 1.433 | 3.166 |
| 13 | 17009.49 | 11.181 | 0.44 | 1.420 | 3.242 |
| 14 | 18034.77 | 10.763 | 3.11 | 1.409 | 3.319 |

| (1)^4^Δ | | | | | |
| --- | --- | --- | --- | --- | --- |
| v | E_v_  (cm^-1^) | B_v_  (cm^-1^) | D_v_ ×10^4^  (cm^-1^) | R_min_  (Å) | R_max_  (Å) |
| 0 | 699.77 | 4.221 | 1.56 | 1.854 | 2.166 |
| 1 | 2072.29 | 4.135 | 1.54 | 1.763 | 2.309 |
| 2 | 3411.30 | 4.049 | 1.53 | 1.706 | 2.420 |
| 3 | 4717.18 | 3.965 | 1.51 | 1.663 | 2.516 |
| 4 | 5990.20 | 8.748 | 1.02 | 1.629 | 2.606 |
| 5 | 7230.81 | 8.722 | 3.01 | 1.599 | 2.692 |
| 6 | 8439.85 | 8.989 | 2.61 | 1.574 | 2.775 |
| 7 | 9617.93 | 9.892 | 1.60 | 1.551 | 2.855 |
| 8 | 10765.69 | 9.235 | 4.80 | 1.531 | 2.934 |
| 9 | 11883.73 | 9.873 | 0.94 | 1.514 | 3.013 |
| 10 | 12972.70 | 9.855 | 2.07 | 1.496 | 3.090 |
| 11 | 14033.20 | 10.183 | 1.18 | 1.481 | 3.168 |
| 12 | 15065.85 | 10.164 | 2.39 | 1.467 | 3.245 |
| 13 | 16071.22 | 10.135 | 4.55 | 1.455 | 3.322 |
| 14 | 17049.89 | 10.087 | 8.15 | 1.443 | 3.400 |

| (2)^4^Δ | | | | | |
| --- | --- | --- | --- | --- | --- |
| v | E_v_  (cm^-1^) | B_v_  (cm^-1^) | D_v_ ×10^4^  (cm^-1^) | R_min_  (Å) | R_max_  (Å) |
| 0 | 632.58 | 4.153 | 1.49 | 1.847 | 2.147 |
| 1 | 2017.36 | 4.137 | 1.53 | 1.753 | 2.297 |
| 2 | 3353.75 | 3.962 | 1.60 | 1.695 | 2.418 |
| 3 | 4634.55 | 8.371 | 6.95 | 1.652 | 2.513 |
| 4 | 5875.58 | 8.614 | 7.51 | 1.619 | 2.604 |
| 5 | 7105.26 | 8.867 | 7.43 | 1.589 | 2.693 |
| 6 | 8270.08 | 9.753 | 0.67 | 1.564 | 2.777 |
| 7 | 9427.03 | 9.417 | 6.27 | 1.542 | 2.860 |
| 8 | 10545.74 | 9.719 | 4.58 | 1.522 | 2.943 |
| 9 | 11620.70 | 9.680 | 1.12 | 1.504 | 3.023 |
| 10 | 12679.36 | 10.005 | 8.67 | 1.487 | 3.104 |
| 11 | 13706.91 | 10.347 | 5.31 | 1.472 | 3.184 |
| 12 | 14695.68 | 10.314 | 10.8 | 1.458 | 3.264 |
| 13 | 15660.88 | 10.254 | 23.3 | 1.445 | 3.345 |
| 14 | 16603.69 | 9.732 | 0.89 | 1.432 | 3.426 |

| (1)^4^Γ | | | | | |
| --- | --- | --- | --- | --- | --- |
| v | E_v_  (cm^-1^) | B_v_  (cm^-1^) | D_v_ ×10^4^  (cm^-1^) | R_min_  (Å) | R_max_  (Å) |
| 0 | 808.36 | 4.746 | 1.66 | 1.750 | 2.041 |
| 1 | 2396.49 | 4.657 | 1.66 | 1.666 | 2.173 |
| 2 | 3945.68 | 4.574 | 1.71 | 1.612 | 2.274 |
| 3 | 5453.60 | 4.500 | 1.81 | 1.570 | 2.362 |
| 4 | 6917.08 | 10.026 | 5.30 | 1.530 | 2.444 |
| 5 | 8338.38 | 10.351 | 5.52 | 1.499 | 2.521 |
| 6 | 9720.18 | 10.285 | 15.70 | 1.472 | 2.594 |
| 7 | 11070.18 | 10.645 | 12.50 | 1.450 | 2.666 |
| 8 | 12391.66 | 10.541 | 29.20 | 1.429 | 2.736 |
| 9 | 13686.11 | 10.961 | 19.90 | 1.411 | 2.806 |
| 10 | 14951.80 | 11.854 | 2.65 | 1.394 | 2.876 |
| 11 | 16186.91 | 11.319 | 24.40 | 1.379 | 2.945 |
| 12 | 17391.54 | 11.792 | 12.40 | 1.365 | 3.014 |
| 13 | 18566.03 | 11.725 | 23.80 | 1.352 | 3.083 |
| 14 | 19711.92 | 11.615 | 42.50 | 1.340 | 3.153 |
| 15 | 20830.68 | 11.439 | 70.70 | 1.330 | 3.222 |
| 16 | 21922.00 | 12.096 | 32.50 | 1.320 | 3.293 |

| (3)^4^Δ | | | | | |
| --- | --- | --- | --- | --- | --- |
| v | E_v_  (cm^-1^) | B_v_  (cm^-1^) | D_v_ ×10^4^  (cm^-1^) | R_min_  (Å) | R_max_  (Å) |
| 0 | 859.31 | 5.061 | 1.78 | 1.694 | 1.976 |
| 1 | 2543.62 | 4.954 | 1.76 | 1.612 | 2.106 |
| 2 | 4185.80 | 4.846 | 1.73 | 1.562 | 2.206 |
| 3 | 5787.22 | 9.687 | 14.50 | 1.524 | 2.294 |
| 4 | 7348.92 | 10.390 | 3.79 | 1.494 | 2.377 |
| 5 | 8871.14 | 10.341 | 10.90 | 1.468 | 2.455 |
| 6 | 10354.21 | 10.238 | 26.60 | 1.446 | 2.530 |
| 7 | 11798.46 | 10.630 | 18.70 | 1.427 | 2.604 |
| 8 | 13205.16 | 10.491 | 39.70 | 1.410 | 2.677 |
| 9 | 14575.40 | 11.457 | 6.21 | 1.395 | 2.748 |
| 10 | 15909.32 | 10.803 | 47.70 | 1.380 | 2.820 |
| 11 | 17207.58 | 11.335 | 25.60 | 1.368 | 2.891 |
| 12 | 18470.75 | 11.201 | 46.90 | 1.356 | 2.962 |
| 13 | 19699.76 | 11.762 | 21.90 | 1.345 | 3.033 |
| 14 | 20894.98 | 11.663 | 38.40 | 1.335 | 3.105 |
| 15 | 22056.99 | 11.509 | 63.10 | 1.325 | 3.176 |
| 16 | 23186.61 | 11.277 | 97.30 | 1.316 | 3.248 |

| (1)^4^Σ^+^ | | | | | |
| --- | --- | --- | --- | --- | --- |
| v | E_v_  (cm^-1^) | B_v_  (cm^-1^) | D_v_ ×10^4^  (cm^-1^) | R_min_  (Å) | R_max_  (Å) |
| 0 | 877.18 | 5.162 | 1.81 | 1.677 | 1.956 |
| 1 | 2594.23 | 5.038 | 1.77 | 1.595 | 2.086 |
| 2 | 4259.41 | 4.885 | 1.73 | 1.552 | 2.190 |
| 3 | 5859.15 | 10.037 | 3.93 | 1.520 | 2.289 |
| 4 | 7378.89 | 10.365 | 3.32 | 1.493 | 2.387 |
| 5 | 8830.81 | 10.324 | 8.96 | 1.470 | 2.474 |
| 6 | 10245.49 | 10.681 | 6.24 | 1.450 | 2.555 |
| 7 | 11632.04 | 10.074 | 44.10 | 1.432 | 2.631 |
| 8 | 12986.46 | 11.020 | 9.03 | 1.415 | 2.705 |
| 9 | 14307.07 | 10.951 | 19.30 | 1.399 | 2.778 |
| 10 | 15591.56 | 11.395 | 10.10 | 1.386 | 2.851 |
| 11 | 16844.86 | 11.330 | 19.90 | 1.373 | 2.923 |
| 12 | 18064.04 | 11.800 | 9.04 | 1.361 | 2.996 |
| 13 | 19252.51 | 11.039 | 62.60 | 1.349 | 3.068 |
| 14 | 20406.27 | 11.666 | 30.10 | 1.339 | 3.140 |

| (1)^4^Φ | | | | | |
| --- | --- | --- | --- | --- | --- |
| v | E_v_  (cm^-1^) | B_v_  (cm^-1^) | D_v_ ×10^4^  (cm^-1^) | R_min_  (Å) | R_max_  (Å) |
| 0 | 762.30 | 4.546 | 1.64 | 1.787 | 2.086 |
| 1 | 2258.38 | 4.455 | 1.62 | 1.700 | 2.223 |
| 2 | 3718.33 | 4.365 | 1.61 | 1.645 | 2.328 |
| 3 | 5142.92 | 4.275 | 1.59 | 1.605 | 2.421 |
| 4 | 6532.69 | 9.439 | 4.59 | 1.571 | 2.507 |
| 5 | 7887.97 | 9.736 | 4.21 | 1.542 | 2.588 |
| 6 | 9209.03 | 9.689 | 11.40 | 1.518 | 2.667 |
| 7 | 10496.64 | 10.383 | 2.44 | 1.496 | 2.743 |
| 8 | 11751.55 | 10.356 | 6.12 | 1.477 | 2.819 |
| 9 | 12974.52 | 10.307 | 14.00 | 1.459 | 2.893 |
| 10 | 14166.25 | 10.217 | 29.00 | 1.444 | 2.967 |
| 11 | 15327.33 | 10.625 | 17.80 | 1.429 | 3.041 |
| 12 | 16458.39 | 10.528 | 34.30 | 1.416 | 3.114 |
| 13 | 17560.06 | 10.982 | 18.80 | 1.403 | 3.188 |
| 14 | 18632.95 | 10.895 | 34.10 | 1.392 | 3.262 |
| 15 | 19677.68 | 11.382 | 16.00 | 1.381 | 3.336 |
| 16 | 20694.83 | 11.317 | 28.10 | 1.371 | 3.410 |

| (1)^4^Π | | | | | |
| --- | --- | --- | --- | --- | --- |
| v | E_v_  (cm^-1^) | B_v_  (cm^-1^) | D_v_ ×10^4^  (cm^-1^) | R_min_  (Å) | R_max_  (Å) |
| 0 | 750.20 | 4.502 | 1.65 | 1.795 | 2.097 |
| 1 | 2221.73 | 4.410 | 1.63 | 1.707 | 2.236 |
| 2 | 3656.92 | 4.318 | 1.61 | 1.653 | 2.342 |
| 3 | 5056.42 | 8.757 | 4.11 | 1.611 | 2.436 |
| 4 | 6420.80 | 9.327 | 1.34 | 1.578 | 2.522 |
| 5 | 7750.42 | 9.297 | 3.98 | 1.550 | 2.605 |
| 6 | 9045.70 | 9.592 | 3.32 | 1.525 | 2.685 |
| 7 | 10307.35 | 10.246 | 0.68 | 1.504 | 2.763 |
| 8 | 11536.16 | 9.867 | 5.89 | 1.484 | 2.840 |
| 9 | 12732.84 | 10.205 | 3.89 | 1.467 | 2.916 |
| 10 | 13898.13 | 10.159 | 8.18 | 1.451 | 2.991 |
| 11 | 15032.69 | 10.079 | 15.90 | 1.436 | 3.066 |
| 12 | 16137.14 | 10.478 | 9.34 | 1.423 | 3.141 |
| 13 | 17212.10 | 10.397 | 16.90 | 1.411 | 3.217 |
| 14 | 18258.21 | 10.833 | 8.82 | 1.399 | 3.292 |
| 15 | 19276.09 | 11.260 | 3.88 | 1.389 | 3.367 |

| (2)^4^Π | | | | | |
| --- | --- | --- | --- | --- | --- |
| v | E_v_  (cm^-1^) | B_v_  (cm^-1^) | D_v_ ×10^4^  (cm^-1^) | R_min_  (Å) | R_max_  (Å) |
| 0 | 676.30 | 4.199 | 1.64 | 1.857 | 2.174 |
| 1 | 2003.44 | 4.118 | 1.66 | 1.763 | 2.320 |
| 2 | 3292.76 | 4.033 | 1.66 | 1.705 | 2.432 |
| 3 | 4546.21 | 3.951 | 1.66 | 1.660 | 2.531 |
| 4 | 5766.51 | 9.167 | 3.00 | 1.624 | 2.622 |
| 5 | 6955.87 | 9.131 | 9.13 | 1.594 | 2.710 |
| 6 | 8113.60 | 9.410 | 8.94 | 1.567 | 2.794 |
| 7 | 9240.75 | 10.050 | 2.51 | 1.544 | 2.876 |
| 8 | 10337.08 | 10.025 | 6.46 | 1.523 | 2.957 |
| 9 | 11403.58 | 10.360 | 4.77 | 1.504 | 3.038 |
| 10 | 12441.08 | 10.326 | 11.10 | 1.486 | 3.118 |
| 11 | 13450.01 | 10.266 | 23.80 | 1.471 | 3.198 |
| 12 | 14431.21 | 10.159 | 46.70 | 1.456 | 3.277 |
| 13 | 15385.25 | 10.581 | 30.40 | 1.442 | 3.357 |

| (2)^4^Φ | | | | | |
| --- | --- | --- | --- | --- | --- |
| v | E_v_  (cm^-1^) | B_v_  (cm^-1^) | D_v_ ×10^4^  (cm^-1^) | R_min_  (Å) | R_max_  (Å) |
| 0 | 622.04 | 4.373 | 2.18 | 1.810 | 2.142 |
| 1 | 1851.21 | 4.304 | 2.12 | 1.711 | 2.290 |
| 2 | 3060.20 | 4.218 | 2.02 | 1.651 | 2.403 |
| 3 | 4248.16 | 9.097 | 13.10 | 1.607 | 2.503 |
| 4 | 5413.77 | 9.385 | 12.0 | 1.573 | 2.596 |
| 5 | 6556.59 | 9.692 | 9.92 | 1.544 | 2.684 |
| 6 | 7674.77 | 9.625 | 23.80 | 1.520 | 2.770 |
| 7 | 8767.28 | 10.351 | 4.62 | 1.498 | 2.854 |
| 8 | 9833.65 | 10.321 | 10.50 | 1.479 | 2.937 |
| 9 | 10873.26 | 10.267 | 21.80 | 1.462 | 3.019 |
| 10 | 11886.06 | 10.174 | 41.50 | 1.447 | 3.101 |
| 11 | 12872.13 | 10.018 | 72.80 | 1.433 | 3.183 |
| 12 | 13831.93 | 10.516 | 41.40 | 1.412 | 3.264 |
| 13 | 14765.91 | 10.385 | 6.910 | 1.407 | 3.346 |

| (1)^4^Σ^−^ | | | | | |
| --- | --- | --- | --- | --- | --- |
| v | E_v_  (cm^-1^) | B_v_  (cm^-1^) | D_v_ ×10^4^  (cm^-1^) | R_min_  (Å) | R_max_  (Å) |
| 0 | 766.86 | 4.559 | 1.63 | 1.785 | 2.083 |
| 1 | 2271.89 | 4.467 | 1.62 | 1.698 | 2.220 |
| 2 | 3740.46 | 4.376 | 1.60 | 1.643 | 2.325 |
| 3 | 5173.16 | 4.286 | 1.59 | 1.603 | 2.417 |
| 4 | 6570.52 | 9.468 | 6.51 | 1.570 | 2.503 |
| 5 | 7932.60 | 9.768 | 5.95 | 1.541 | 2.584 |
| 6 | 9259.80 | 9.712 | 16.20 | 1.517 | 2.663 |
| 7 | 10552.76 | 10.421 | 3.48 | 1.495 | 2.739 |
| 8 | 11812.24 | 10.390 | 8.69 | 1.476 | 2.815 |
| 9 | 13039.03 | 10.331 | 19.80 | 1.458 | 2.890 |
| 10 | 14233.76 | 10.222 | 40.80 | 1.443 | 2.964 |
| 11 | 15397.10 | 10.647 | 25.30 | 1.428 | 3.038 |
| 12 | 16529.54 | 10.532 | 48.30 | 1.415 | 3.112 |
| 13 | 17631.81 | 11.007 | 26.70 | 1.403 | 3.186 |
| 14 | 18704.53 | 10.903 | 48.40 | 1.391 | 3.260 |
| 15 | 19748.36 | 11.413 | 22.90 | 1.381 | 3.335 |
| 16 | 20763.89 | 11.336 | 40.00 | 1.370 | 3.410 |

| (4)^4^Δ | | | | | |
| --- | --- | --- | --- | --- | --- |
| v | E_v_  (cm^-1^) | B_v_  (cm^-1^) | D_v_ ×10^4^  (cm^-1^) | R_min_  (Å) | R_max_  (Å) |
| 0 | 699.16 | 4.222 | 1.57 | 1.854 | 2.166 |
| 1 | 2068.42 | 4.142 | 1.61 | 1.763 | 2.310 |
| 2 | 3397.12 | 4.075 | 1.72 | 1.702 | 2.418 |
| 3 | 4682.03 | 4.019 | 1.84 | 1.650 | 2.514 |
| 4 | 5924.50 | 8.999 | 1.33 | 1.612 | 2.602 |
| 5 | 7132.19 | 9.274 | 1.31 | 1.580 | 2.685 |
| 6 | 8314.00 | 9.247 | 3.49 | 1.554 | 2.766 |
| 7 | 9473.06 | 9.542 | 2.71 | 1.530 | 2.845 |
| 8 | 10607.29 | 9.852 | 1.95 | 1.510 | 2.923 |
| 9 | 11713.87 | 9.822 | 4.41 | 1.491 | 3.001 |
| 10 | 12792.03 | 10.155 | 2.77 | 1.474 | 3.077 |
| 11 | 13843.07 | 10.121 | 5.61 | 1.459 | 3.154 |
| 12 | 14868.60 | 10.862 | 0.786 | 1.445 | 3.230 |
| 13 | 15869.31 | 9.966 | 18.30 | 1.432 | 3.307 |

| (2)^4^Σ^−^ | | | | | |
| --- | --- | --- | --- | --- | --- |
| v | E_v_  (cm^-1^) | B_v_  (cm^-1^) | D_v_ ×10^4^  (cm^-1^) | R_min_  (Å) | R_max_  (Å) |
| 0 | 770.73 | 4.590 | 1.62 | 1.776 | 2.076 |
| 1 | 2292.13 | 4.474 | 1.48 | 1.696 | 2.214 |
| 2 | 3793.23 | 4.349 | 1.38 | 1.649 | 2.320 |
| 3 | 5267.21 | 8.777 | 2.21 | 1.612 | 2.415 |
| 4 | 6703.40 | 9.339 | 0.662 | 1.581 | 2.503 |
| 5 | 8094.61 | 9.318 | 2.04 | 1.554 | 2.586 |
| 6 | 9441.15 | 9.612 | 1.69 | 1.530 | 2.666 |
| 7 | 10747.96 | 9.920 | 1.23 | 1.509 | 2.744 |
| 8 | 12018.76 | 9.896 | 2.99 | 1.490 | 2.821 |
| 9 | 13254.00 | 10.227 | 1.93 | 1.473 | 2.898 |
| 10 | 14452.28 | 10.198 | 4.25 | 1.458 | 2.974 |
| 11 | 15612.98 | 10.147 | 8.69 | 1.443 | 3.051 |
| 12 | 16736.16 | 10.059 | 16.40 | 1.430 | 3.128 |
| 13 | 17822.77 | 10.471 | 9.37 | 1.418 | 3.206 |
| 14 | 18873.42 | 10.388 | 16.60 | 1.407 | 3.286 |
| 15 | 19888.99 | 10.833 | 8.35 | 1.396 | 3.366 |

| (5)^4^Δ | | | | | |
| --- | --- | --- | --- | --- | --- |
| v | E_v_  (cm^-1^) | B_v_  (cm^-1^) | D_v_ ×10^4^  (cm^-1^) | R_min_  (Å) | R_max_  (Å) |
| 0 | 693.82 | 4.269 | 1.64 | 1.842 | 2.155 |
| 1 | 2051.84 | 4.173 | 1.64 | 1.751 | 2.301 |
| 2 | 3370.62 | 4.078 | 1.63 | 1.964 | 2.414 |
| 3 | 4651.18 | 3.986 | 1.61 | 1.652 | 2.514 |
| 4 | 5896.78 | 9.461 | 0.72 | 1.618 | 2.606 |
| 5 | 7109.86 | 9.132 | 7.33 | 1.590 | 2.694 |
| 6 | 8289.78 | 9.418 | 6.49 | 1.564 | 2.779 |
| 7 | 9436.99 | 9.721 | 5.18 | 1.542 | 2.862 |
| 8 | 10552.12 | 9.678 | 12.60 | 1.521 | 2.943 |
| 9 | 11635.86 | 10.370 | 2.53 | 1.503 | 3.024 |
| 10 | 12688.89 | 9.952 | 19.40 | 1.487 | 3.105 |
| 11 | 13712.16 | 10.313 | 12.40 | 1.472 | 3.185 |
| 12 | 14706.30 | 10.251 | 24.70 | 1.458 | 3.266 |
| 13 | 15672.16 | 10.645 | 14.30 | 1.445 | 3.346 |

Table (TS2): The rovibrational constants for the different vibrational levels of different

electronic states of the spin-orbit coupling ZrH molecule.

| X^2^Δ_5/2_ | | | | | |
| --- | --- | --- | --- | --- | --- |
| v | E_v_  (cm^-1^) | B_v_  (cm^-1^) | D_v_ ×10^4^  (cm^-1^) | R_min_  (Å) | R_max_  (Å) |
| 0 | 815.91 | 4.659 | 1.54 | 1.769 | 2.054 |
| 1 | 2412.54 | 4.542 | 1.71 | 1.686 | 2.196 |
| 2 | 3910.72 | 4.411 | 1.79 | 1.634 | 2.307 |
| 3 | 5339.73 | 9.450 | 17.7 | 1.588 | 2.401 |
| 4 | 6686.59 | 10.106 | 3.05 | 1.553 | 2.510 |
| 5 | 7932.11 | 10.058 | 17.5 | 1.525 | 2.602 |
| 6 | 9151.24 | 10.402 | 11.9 | 1.501 | 2.674 |
| 7 | 10401.30 | 10.359 | 18.6 | 1.478 | 2.748 |

| (1)^4^φ_5/2_ | | | | | |
| --- | --- | --- | --- | --- | --- |
| V | E_v_  (cm^-1^) | B_v_  (cm^-1^) | D_v_ ×10^4^  (cm^-1^) | R_min_  (Å) | R_max_  (Å) |
| 0 | 825.95 | 4.640 | 1.59 | 1.777 | 2.059 |
| 1 | 2395.67 | 4.567 | 2.01 | 1.678 | 2.200 |
| 2 | 3836.87 | 9.059 | 4.018 | 1.618 | 2.307 |
| 3 | 5220.65 | 9.3322 | 4.69 | 1.576 | 2.398 |
| 4 | 6535.27 | 9.6218 | 5.61 | 1.543 | 2.496 |

| (1)^4^Φ_7/2_ | | | | | |
| --- | --- | --- | --- | --- | --- |
| V | E_v_  (cm^-1^) | B_v_  (cm^-1^) | D_v_ ×10^4^  (cm^-1^) | R_min_  (Å) | R_max_  (Å) |
| 0 | 862.46 | 4.652 | 1.62 | 1.771 | 2.049 |
| 1 | 2418.48 | 4.552 | 2.03 | 1.679 | 2.204 |
| 2 | 3840.19 | 4.454 | 1.39 | 1.623 | 2.308 |
| 3 | 5276.94 | 9.027 | 3.57 | 1.586 | 2.401 |
| 4 | 6639.77 | 9.304 | 3.45 | 1.556 | 2.494 |
| 5 | 7912.81 | 9.598 | 3.34 | 1.531 | 2.606 |
| 6 | 9137.76 | 9.910 | 2.11 | 1.510 | 2.679 |
| 7 | 10381.53 | 10.235 | 1.43 | 1.490 | 2.753 |

| (1)^4^Σ^-^_1/2_ | | | | | |
| --- | --- | --- | --- | --- | --- |
| V | E_v_  (cm^-1^) | B_v_  (cm^-1^) | D_v_ ×10^4^  (cm^-1^) | R_min_  (Å) | R_max_  (Å) |
| 0 | 749.64 | 4.5801 | 1.70 | 1.780 | 2.084 |
| 1 | 2240.98 | 4.530 | 1.65 | 1.685 | 2.209 |
| 2 | 3714.52 | 4.434 | 1.71 | 1.632 | 2.314 |
| 3 | 5133.77 | 9.148 | 7.06 | 1.594 | 2.407 |
| 4 | 6495.84 | 9.431 | 7.30 | 1.563 | 2.503 |
|  |  |  |  |  |  |

| (1)^4^Σ^−^_3/2_ | | | | | |
| --- | --- | --- | --- | --- | --- |
| V | E_v_  (cm^-1^) | B_v_  (cm^-1^) | D_v_ ×10^4^  (cm^-1^) | R_min_  (Å) | R_max_  (Å) |
| 0 | 752.10 | 4.585 | 1.66 | 1.778 | 2.081 |
| 1 | 2268.29 | 4.543 | 1.46 | 1.689 | 2.204 |
| 2 | 3810.30 | 4.481 | 1.48 | 1.635 | 2.294 |
| 3 | 5320.07 | 9.201 | 4.76 | 1.594 | 2.396 |
| 4 | 6675.37 | 9.477 | 9.55 | 1.563 | 2.513 |
| 5 | 7932.76 | 9.794 | 3.18 | 1.538 | 2.601 |
| 6 | 9209.42 | 9.736 | 12.6 | 1.515 | 2.677 |
| 7 | 10457.13 | 10.439 | 4.74 | 1.494 | 2.751 |
| 8 | 11635.25 | 10.418 | 7.17 | 1.476 | 2.867 |
| 9 | 12733.53 | 10.378 | 12.4 | 1.461 | 2.934 |
| 10 | 13873.21 | 10.262 | 36.4 | 1.446 | 3.005 |
| 11 | 15016.00 | 10.679 | 26.8 | 1.431 | 3.078 |
| 12 | 16096.70 | 10.618 | 34.6 | 1.418 | 3.149 |
| 13 | 17175.85 | 11.071 | 15.0 | 1.406 | 3.221 |
| 14 | 18224.10 | 10.959 | 42.1 | 1.395 | 3.294 |
| 15 | 19245.32 | 11.452 | 22.0 | 1.385 | 3.367 |

| (1)^4^Φ_9/2_ | | | | | |
| --- | --- | --- | --- | --- | --- |
| V | E_v_  (cm^-1^) | B_v_  (cm^-1^) | D_v_ ×10^4^  (cm^-1^) | R_min_  (Å) | R_max_  (Å) |
| 0 | 818.61 | 4.599 | 1.43 | 1.783 | 2.070 |
| 1 | 2466.54 | 4.582 | 1.49 | 1.691 | 2.187 |
| 2 | 4067.61 | 4.456 | 1.86 | 1.635 | 2.291 |
| 3 | 5533.68 | 9.102 | 3.80 | 1.595 | 2.395 |
| 4 | 6854.04 | 9.381 | 4.45 | 1.565 | 2.529 |
| 5 | 8083.14 | 9.680 | 3.50 | 1.540 | 2.610 |
| 6 | 9340.06 | 9.642 | 7.72 | 1.517 | 2.685 |
| 7 | 10598.30 | 10.322 | 1.75 | 1.496 | 2.761 |
| 8 | 11773.45 | 10.300 | 4.26 | 1.478 | 2.858 |
| 9 | 12918.62 | 10.263 | 9.09 | 1.461 | 2.928 |
| 10 | 14068.56 | 10.200 | 16.6 | 1.446 | 3.000 |
| 11 | 15212.89 | 10.587 | 10.5 | 1.432 | 3.072 |
| 12 | 16303.58 | 10.518 | 19.6 | 1.419 | 3.144 |
| 13 | 17388.26 | 10.406 | 34.3 | 1.407 | 3.216 |
| 14 | 18439.48 | 10.882 | 19.2 | 1.395 | 3.289 |
| 15 | 19466.78 | 11.338 | 8.08 | 1.385 | 3.362 |

| (1)^2^Π_3/2_ | | | | | |
| --- | --- | --- | --- | --- | --- |
| V | E_v_  (cm^-1^) | B_v_  (cm^-1^) | D_v_ ×10^4^  (cm^-1^) | R_min_  (Å) | R_max_  (Å) |
| 0 | 862.67 | 4.812 | 1.52 | 1.740 | 2.023 |
| 1 | 2544.15 | 4.671 | 1.53 | 1.664 | 2.160 |
| 2 | 4156.33 | 8.869 | 10.1 | 1.614 | 2.262 |
| 3 | 5655.33 | 9.461 | 4.42 | 1.577 | 2.384 |
| 4 | 6975.11 | 9.409 | 14.8 | 1.549 | 2.500 |
| 5 | 8250.42 | 9.722 | 10.0 | 1.525 | 2.579 |
| 6 | 9559.02 | 10.045 | 7.87 | 1.503 | 2.659 |
| 7 | 10822.50 | 9.983 | 19.3 | 1.483 | 2.744 |
| 8 | 12040.58 | 10.344 | 12.3 | 1.465 | 2.820 |
| 9 | 13241.47 | 10.268 | 26.0 | 1.449 | 2.895 |
| 10 | 14417.23 | 10.665 | 16.2 | 1.434 | 2.971 |
| 11 | 15558.53 | 10.584 | 31.4 | 1.421 | 3.046 |
| 12 | 16669.34 | 11.025 | 16.4 | 1.408 | 3.121 |
| 13 | 17755.15 | 10.949 | 31.7 | 1.397 | 3.196 |
| 14 | 18809.11 | 10.831 | 53.9 | 1.386 | 3.271 |
| 15 | 19833.93 | 11.366 | 25.9 | 1.375 | 3.347 |
| 16 | 20834.08 | 11.280 | 43.5 | 1.366 | 3.423 |

| (1)^4^Π_1_ | | | | | |
| --- | --- | --- | --- | --- | --- |
| V | E_v_  (cm^-1^) | B_v_  (cm^-1^) | D_v_ ×10^4^  (cm^-1^) | R_min_  (Å) | R_max_  (Å) |
| 0 | 755.58 | 4.510 | 1.63 | 1.794 | 2.095 |
| 1 | 2236.72 | 4.417 | 1.62 | 1.706 | 2.233 |
| 2 | 3680.41 | 4.322 | 1.65 | 1.652 | 2.339 |
| 3 | 5075.70 | 8.777 | 4.81 | 1.611 | 2.437 |
| 4 | 6410.53 | 9.658 | 0.45 | 1.579 | 2.534 |
| 5 | 7702.07 | 9.322 | 4.45 | 1.551 | 2.613 |
| 6 | 8974.29 | 9.619 | 3.69 | 1.527 | 2.693 |
| 7 | 10174.66 | 9.932 | 3.00 | 1.507 | 2.802 |
| 8 | 11310.58 | 9.900 | 6.31 | 1.489 | 2.871 |
| 9 | 12476.23 | 10.605 | 0.94 | 1.471 | 2.943 |
| 10 | 13637.33 | 10.200 | 8.06 | 1.445 | 3.016 |
| 11 | 14742.57 | 10.565 | 4.61 | 1.441 | 3.088 |
| 12 | 15841.79 | 10.017 | 27.2 | 1.427 | 3.161 |
| 13 | 16906.28 | 11.332 | 1.05 | 1.415 | 3.225 |
| 14 | 17946.67 | 10.347 | 27.3 | 1.404 | 3.308 |

| (1)^4^Π_1/2_ | | | | | |
| --- | --- | --- | --- | --- | --- |
| V | E_v_  (cm^-1^) | B_v_  (cm^-1^) | D_v_ ×10^4^  (cm^-1^) | R_min_  (Å) | R_max_  (Å) |
| 0 | 766.99 | 4.514 | 1.60 | 1.794 | 2.091 |
| 1 | 2268.53 | 4.428 | 1.61 | 1.706 | 2.230 |
| 2 | 3727.41 | 4.336 | 1.61 | 1.651 | 2.330 |
| 3 | 5141.38 | 8.759 | 4.14 | 1.610 | 2.437 |
| 4 | 6481.74 | 9.330 | 1.36 | 1.578 | 2.525 |
| 5 | 7795.62 | 9.301 | 3.80 | 1.555 | 2.606 |
| 6 | 9075.61 | 9.597 | 3.39 | 1.526 | 2.690 |
| 7 | 10257.69 | 10.251 | 0.72 | 1.506 | 2.802 |
| 8 | 11387.99 | 9.878 | 5.52 | 1.488 | 2.870 |
| 9 | 12558.30 | 10.217 | 3.05 | 1.471 | 2.941 |
| 10 | 13726.57 | 10.177 | 7.11 | 1.455 | 3.014 |
| 11 | 14831.50 | 10.540 | 3.90 | 1.441 | 3.084 |
| 12 | 15942.67 | 10.001 | 23.5 | 1.427 | 3.157 |
| 13 | 17009.95 | 10.891 | 3.93 | 1.415 | 3.229 |
| 14 | 18062.91 | 10.327 | 23.4 | 1.403 | 3.302 |

| (1)^4^Π_−1/2_ | | | | | |
| --- | --- | --- | --- | --- | --- |
| V | E_v_  (cm^-1^) | B_v_  (cm^-1^) | D_v_ ×10^4^  (cm^-1^) | R_min_  (Å) | R_max_  (Å) |
| 0 | 766.46 | 4.516 | 1.56 | 1.795 | 2.093 |
| 1 | 2292.55 | 4.460 | 1.47 | 1.705 | 2.219 |
| 2 | 3813.40 | 4.387 | 1.70 | 1.649 | 2.315 |
| 3 | 5248.17 | 8.749 | 4.47 | 1.608 | 2.486 |
| 4 | 6534.90 | 9.322 | 1.43 | 1.577 | 2.531 |
| 5 | 7834.22 | 9.294 | 3.65 | 1.550 | 2.611 |
| 6 | 9119.12 | 9.589 | 3.13 | 1.526 | 2.692 |
| 7 | 10345.78 | 10.241 | 0.64 | 1.505 | 2.780 |
| 8 | 11533.91 | 9.868 | 5.28 | 1.486 | 2.855 |
| 9 | 12714.63 | 10.205 | 3.25 | 1.469 | 2.930 |
| 10 | 13871.16 | 10.164 | 7.07 | 1.453 | 3.005 |
| 11 | 14988.78 | 10.094 | 13.6 | 1.439 | 3.079 |
| 12 | 16085.48 | 10.486 | 7.67 | 1.425 | 3.154 |
| 13 | 17148.30 | 10.415 | 14.3 | 1.413 | 3.229 |
| 14 | 18188.59 | 10.305 | 23.9 | 1.402 | 3.305 |

| (1)^4^Δ_1/2_ | | | | | |
| --- | --- | --- | --- | --- | --- |
| V | E_v_  (cm^-1^) | B_v_  (cm^-1^) | D_v_ ×10^4^  (cm^-1^) | R_min_  (Å) | R_max_  (Å) |
| 0 | 680.37 | 4.228 | 1.84 | 1.850 | 2.164 |
| 1 | 1937.91 | 4.093 | 2.16 | 1.750 | 2.339 |
| 2 | 3097.64 | 4.007 | 1.41 | 1.695 | 2.459 |
| 3 | 4299.89 | 8.483 | 2.83 | 1.653 | 2.533 |
| 4 | 5561.65 | 9.018 | 0.85 | 1.617 | 2.600 |
| 5 | 6814.95 | 8.994 | 2.79 | 1.586 | 2.709 |
| 6 | 7976.98 | 9.273 | 2.64 | 1.562 | 2.786 |
| 7 | 9109.50 | 9.888 | 0.47 | 1.539 | 2.853 |
| 8 | 10283.23 | 9.539 | 3.99 | 1.518 | 2.928 |
| 9 | 11410.08 | 9.852 | 2.97 | 1.499 | 3.001 |
| 10 | 12506.44 | 10.179 | 1.71 | 1.482 | 3.073 |
| 11 | 13594.13 | 10.153 | 3.84 | 1.467 | 3.147 |
| 12 | 14639.23 | 10.112 | 7.18 | 1.452 | 3.220 |
| 13 | 15673.33 | 10.039 | 13.8 | 1.439 | 3.295 |
| 14 | 16669.29 | 10.436 | 7.32 | 1.426 | 3.369 |

| (1)^4^Δ_5/2_ | | | | | |
| --- | --- | --- | --- | --- | --- |
| V | E_v_  (cm^-1^) | B_v_  (cm^-1^) | D_v_ ×10^4^  (cm^-1^) | R_min_  (Å) | R_max_  (Å) |
| 0 | 688.12 | 4.199 | 1.74 | 1.854 | 2.169 |
| 1 | 1958.13 | 4.030 | 1.79 | 1.768 | 2.350 |
| 2 | 3174.73 | 3.994 | 1.27 | 1.714 | 2.455 |
| 3 | 4449.56 | 8.501 | 2.47 | 1.662 | 2.522 |
| 4 | 5738.26 | 9.325 | 3.02 | 1.621 | 2.598 |
| 5 | 6939.77 | 9.009 | 3.95 | 1.588 | 2.728 |
| 6 | 8018.66 | 9.290 | 3.20 | 1.563 | 2.795 |
| 7 | 9153.27 | 9.587 | 1.90 | 1.541 | 2.865 |
| 8 | 10317.75 | 9.556 | 5.04 | 1.519 | 2.939 |
| 9 | 11418.76 | 10.219 | 9.64 | 1.500 | 3.010 |
| 10 | 12515.62 | 10.203 | 2.07 | 1.483 | 3.083 |
| 11 | 13586.66 | 10.175 | 4.64 | 1.467 | 3.156 |
| 12 | 14626.25 | 10.131 | 8.64 | 1.453 | 3.230 |
| 13 | 15648.19 | 10.052 | 16.7 | 1.439 | 3.307 |
| 14 | 16639.60 | 10.458 | 8.91 | 1.427 | 3.378 |

| (1)^4^Δ_3/2_ | | | | | |
| --- | --- | --- | --- | --- | --- |
| V | E_v_  (cm^-1^) | B_v_  (cm^-1^) | D_v_ ×10^4^  (cm^-1^) | R_min_  (Å) | R_max_  (Å) |
| 0 | 723.96 | 4.245 | 1.71 | 1.847 | 2.155 |
| 1 | 2024.80 | 4.057 | 1.76 | 1.763 | 2.333 |
| 2 | 3264.50 | 4.010 | 1.47 | 1.705 | 2.443 |
| 3 | 4524.19 | 8.462 | 2.01 | 1.662 | 2.524 |
| 4 | 5803.54 | 8.709 | 2.24 | 1.621 | 2.600 |
| 5 | 7035.77 | 8.969 | 2.51 | 1.588 | 2.697 |
| 6 | 8214.81 | 9.247 | 2.18 | 1.561 | 2.764 |
| 7 | 9397.60 | 9.540 | 1.58 | 1.536 | 2.842 |
| 8 | 10566.57 | 9.508 | 4.19 | 1.515 | 2.921 |
| 9 | 11666.80 | 10.165 | 0.79 | 1.496 | 2.995 |
| 10 | 12772.20 | 10.149 | 1.67 | 1.478 | 3.072 |
| 11 | 13835.58 | 10.123 | 3.55 | 1.463 | 3.147 |
| 12 | 14882.96 | 10.080 | 6.63 | 1.449 | 3.222 |
| 13 | 15896.95 | 10.448 | 3.69 | 1.436 | 3.297 |
| 14 | 16891.72 | 10.406 | 6.57 | 1.424 | 3.372 |

| (1)^4^Δ_7/2_ | | | | | |
| --- | --- | --- | --- | --- | --- |
| V | E_v_  (cm^-1^) | B_v_  (cm^-1^) | D_v_ ×10^4^  (cm^-1^) | R_min_  (Å) | R_max_  (Å) |
| 0 | 727.20 | 4.230 | 1.72 | 1.851 | 2.158 |
| 1 | 2020.23 | 4.049 | 1.90 | 1.762 | 2.340 |
| 2 | 3235.06 | 4.028 | 1.31 | 1.701 | 2.445 |
| 3 | 4501.71 | 8.291 | 7.12 | 1.660 | 2.522 |
| 4 | 5789.01 | 9.110 | 0.72 | 1.626 | 2.606 |
| 5 | 7027.15 | 8.786 | 7.22 | 1.597 | 2.694 |
| 6 | 8241.42 | 9.060 | 5.92 | 1.573 | 2.770 |
| 7 | 9447.63 | 9.671 | 1.40 | 1.545 | 2.844 |
| 8 | 10618.31 | 9.649 | 3.64 | 1.524 | 2.922 |
| 9 | 11751.49 | 9.961 | 2.64 | 1.507 | 3.000 |
| 10 | 12844.16 | 9.935 | 5.41 | 1.491 | 3.076 |
| 11 | 13922.43 | 10.267 | 3.47 | 1.476 | 3.153 |
| 12 | 14967.47 | 10.238 | 6.82 | 1.462 | 3.229 |

| (2)^4^Σ^−^_1/2_ | | | | | |
| --- | --- | --- | --- | --- | --- |
| V | E_v_  (cm^-1^) | B_v_  (cm^-1^) | D_v_ ×10^4^  (cm^-1^) | R_min_  (Å) | R_max_  (Å) |
| 0 | 700.51 | 4.497 | 2.07 | 1.782 | 2.090 |
| 1 | 1965.63 | 4.209 | 1.85 | 1.713 | 2.303 |
| 2 | 3196.16 | 8.226 | 3.34 | 1.669 | 2.385 |
| 3 | 4539.45 | 8.739 | 1.20 | 1.631 | 2.469 |
| 4 | 5696.23 | 8.711 | 5.00 | 1.604 | 2.648 |
| 5 | 6752.68 | 9.581 | 2.12 | 1.581 | 2.706 |
| 6 | 7982.22 | 9.265 | 1.62 | 1.557 | 2.773 |
| 7 | 9211.66 | 9.557 | 1.35 | 1.534 | 2.841 |
| 8 | 10382.25 | 9.533 | 2.84 | 1.515 | 2.906 |
| 9 | 11575.87 | 9.842 | 1.93 | 1.497 | 2.975 |
| 10 | 12725.82 | 10.163 | 1.18 | 1.481 | 3.043 |
| 11 | 13865.26 | 10.141 | 2.52 | 1.465 | 3.111 |
| 12 | 14976.53 | 10.106 | 4.78 | 1.451 | 3.179 |
| 13 | 16067.36 | 10.045 | 8.82 | 1.438 | 3.246 |
| 14 | 17137.94 | 10.430 | 4.87 | 1.426 | 3.317 |

| (2)^4^Σ^−^_3/2_ | | | | | |
| --- | --- | --- | --- | --- | --- |
| V | E_v_  (cm^-1^) | B_v_  (cm^-1^) | D_v_ ×10^4^  (cm^-1^) | R_min_  (Å) | R_max_  (Å) |
| 0 | 773.31 | 4.567 | 1.62 | 1.778 | 2.076 |
| 1 | 2271.03 | 4.404 | 1.63 | 1.704 | 2.231 |
| 2 | 3696.19 | 8.208 | 3.61 | 1.654 | 2.337 |
| 3 | 5023.55 | 8.723 | 1.93 | 1.620 | 2.540 |
| 4 | 6010.87 | 8.691 | 6.52 | 1.597 | 2.649 |
| 5 | 7087.12 | 8.971 | 2.37 | 1.574 | 2.707 |
| 6 | 8325.74 | 9.251 | 1.83 | 1.552 | 2.774 |
| 7 | 9524.69 | 9.544 | 1.26 | 1.532 | 2.840 |
| 8 | 10720.43 | 9.519 | 2.90 | 1.513 | 2.907 |
| 9 | 11895.96 | 9.829 | 1.79 | 1.495 | 2.974 |
| 10 | 13059.77 | 10.153 | 1.16 | 1.479 | 3.042 |
| 11 | 14189.80 | 10.132 | 2.23 | 1.464 | 3.110 |
| 12 | 15308.35 | 10.096 | 4.57 | 1.450 | 3.179 |
| 13 | 16395.66 | 10.037 | 7.90 | 1.437 | 3.248 |
| 14 | 17461.06 | 10.419 | 4.48 | 1.425 | 3.318 |

| (1)^2^Σ^−^_1/2_ | | | | | |
| --- | --- | --- | --- | --- | --- |
| V | E_v_  (cm^-1^) | B_v_  (cm^-1^) | D_v_ ×10^4^  (cm^-1^) | R_min_  (Å) | R_max_  (Å) |
| 0 | 796.90 | 4.553 | 1.53 | 1.788 | 2.079 |
| 1 | 2348.86 | 4.451 | 1.57 | 1.702 | 2.220 |
| 2 | 3837.49 | 4.328 | 1.49 | 1.652 | 2.324 |
| 3 | 5273.63 | 8.865 | 3.99 | 1.615 | 2.446 |
| 4 | 6609.01 | 9.745 | 0.31 | 1.585 | 2.530 |
| 5 | 7930.87 | 9.089 | 9.37 | 1.559 | 2.610 |
| 6 | 9261.92 | 9.380 | 8.00 | 1.539 | 2.691 |
| 7 | 10529.14 | 9.688 | 6.04 | 1.518 | 2.768 |
| 8 | 11787.98 | 10.008 | 4.10 | 1.499 | 2.845 |
| 9 | 12998.13 | 10.342 | 2.70 | 1.482 | 2.920 |
| 10 | 14189.50 | 10.315 | 6.09 | 1.466 | 2.996 |

| (2)^2^Δ_3/2_ | | | | | |
| --- | --- | --- | --- | --- | --- |
| V | E_v_  (cm^-1^) | B_v_  (cm^-1^) | D_v_ ×10^4^  (cm^-1^) | R_min_  (Å) | R_max_  (Å) |
| 0 | 714.80 | 4.493 | 1.86 | 1.791 | 2.099 |
| 1 | 2069.69 | 4.274 | 2.57 | 1.709 | 2.262 |
| 2 | 3198.91 | 8.484 | 4.91 | 1.663 | 2.441 |
| 3 | 4335.81 | 9.024 | 9.86 | 1.627 | 2.510 |
| 4 | 5632.44 | 9.001 | 2.74 | 1.592 | 2.586 |
| 5 | 6887.03 | 9.278 | 2.60 | 1.563 | 2.660 |
| 6 | 8120.28 | 9.894 | 0.56 | 1.538 | 2.733 |
| 7 | 9349.24 | 9.538 | 4.97 | 1.516 | 2.807 |
| 8 | 10537.77 | 9.853 | 3.52 | 1.496 | 2.880 |
| 9 | 11708.75 | 10.182 | 2.33 | 1.478 | 2.954 |
| 10 | 12837.79 | 10.151 | 4.98 | 1.461 | 3.028 |
| 11 | 13933.99 | 10.094 | 10.5 | 1.446 | 3.117 |
| 12 | 14978.21 | 10.000 | 19.2 | 1.433 | 3.194 |
| 13 | 15988.83 | 10.418 | 10.7 | 1.421 | 3.271 |
| 14 | 16985.84 | 10.332 | 18.2 | 1.409 | 3.352 |

| (2)^2^Σ^−^_1/2_ | | | | | |
| --- | --- | --- | --- | --- | --- |
| V | E_v_  (cm^-1^) | B_v_  (cm^-1^) | D_v_ ×10^4^  (cm^-1^) | R_min_  (Å) | R_max_  (Å) |
| 0 | 743.39 | 4.488 | 1.78 | 1.798 | 2.098 |
| 1 | 2126.58 | 4.275 | 2.65 | 1.709 | 2.520 |
| 2 | 3250.99 | 8.517 | 6.38 | 1.664 | 2.446 |
| 3 | 4362.69 | 9.652 | 0.10 | 1.628 | 2.513 |
| 4 | 5657.10 | 9.039 | 3.20 | 1.594 | 2.599 |
| 5 | 6910.45 | 9.318 | 3.07 | 1.565 | 2.664 |
| 6 | 8141.40 | 9.938 | 0.67 | 1.540 | 2.737 |
| 7 | 9366.81 | 9.578 | 5.98 | 1.518 | 2.810 |
| 8 | 10553.73 | 10.247 | 1.19 | 1.497 | 2.883 |
| 9 | 11724.75 | 10.228 | 2.81 | 1.479 | 2.957 |
| 10 | 12856.12 | 10.195 | 6.15 | 1.463 | 3.030 |
| 11 | 13959.01 | 10.136 | 12.9 | 1.448 | 3.114 |
| 12 | 15014.00 | 10.518 | 7.37 | 1.434 | 3.189 |
| 13 | 16037.55 | 10.460 | 13.9 | 1.422 | 3.267 |
| 14 | 17039.88 | 10.875 | 6.86 | 1.410 | 3.356 |

| (1)^2^Γ_7/2_ | | | | | |
| --- | --- | --- | --- | --- | --- |
| V | E_v_  (cm^-1^) | B_v_  (cm^-1^) | D_v_ ×10^4^  (cm^-1^) | R_min_  (Å) | R_max_  (Å) |
| 0 | 758.12 | 4.539 | 1.71 | 1.788 | 2.089 |
| 1 | 2206.85 | 4.392 | 1.79 | 1.703 | 2.235 |
| 2 | 3581.20 | 8.487 | 4.12 | 1.650 | 2.357 |
| 3 | 4902.20 | 9.028 | 1.49 | 1.611 | 2.451 |
| 4 | 6214.31 | 9.304 | 1.42 | 1.578 | 2.546 |
| 5 | 7462.06 | 9.275 | 4.09 | 1.551 | 2.637 |

| (2)^4^Δ_1/2_ | | | | | |
| --- | --- | --- | --- | --- | --- |
| V | E_v_  (cm^-1^) | B_v_  (cm^-1^) | D_v_ ×10^4^  (cm^-1^) | R_min_  (Å) | R_max_  (Å) |
| 0 | 687.34 | 4.255 | 1.68 | 1.843 | 2.157 |
| 1 | 2020.80 | 4.150 | 1.59 | 1.754 | 2.308 |
| 2 | 3339.55 | 4.101 | 1.43 | 1.697 | 2.413 |
| 3 | 4662.18 | 8.572 | 9.06 | 1.653 | 2.505 |
| 4 | 5931.11 | 9.433 | 1.27 | 1.618 | 2.592 |
| 5 | 7178.99 | 9.408 | 4.47 | 1.587 | 2.681 |
| 6 | 8360.35 | 9.354 | 13.6 | 1.556 | 2.765 |
| 7 | 9516.39 | 10.365 | 1.06 | 1.530 | 2.848 |
| 8 | 10625.48 | 9.981 | 10.0 | 1.507 | 2.928 |
| 9 | 11710.54 | 10.323 | 7.32 | 1.487 | 3.008 |
| 10 | 12765.81 | 10.274 | 15.8 | 1.470 | 3.084 |
| 11 | 13806.25 | 10.187 | 31.6 | 1.453 | 3.152 |
| 12 | 14829.06 | 10.598 | 19.3 | 1.439 | 3.233 |
| 13 | 15812.22 | 10.506 | 37.9 | 1.425 | 3.314 |

| (2)^4^Δ_3/2_ | | | | | |
| --- | --- | --- | --- | --- | --- |
| V | E_v_  (cm^-1^) | B_v_  (cm^-1^) | D_v_ ×10^4^  (cm^-1^) | R_min_  (Å) | R_max_  (Å) |
| 0 | 683.05 | 4.247 | 1.75 | 1.843 | 2.161 |
| 1 | 1985.48 | 4.131 | 1.58 | 1.753 | 2.319 |
| 2 | 3288.85 | 4.104 | 1.43 | 1.694 | 2.418 |
| 3 | 4603.52 | 8.567 | 10.4 | 1.653 | 2.512 |
| 4 | 5873.42 | 9.436 | 1.41 | 1.616 | 2.600 |
| 5 | 7111.79 | 9.410 | 4.97 | 1.581 | 2.682 |
| 6 | 8321.40 | 9.352 | 14.4 | 1.551 | 2.759 |
| 7 | 9494.86 | 10.367 | 1.15 | 1.526 | 2.835 |
| 8 | 10629.49 | 9.978 | 11.2 | 1.504 | 2.915 |
| 9 | 11717.21 | 10.321 | 7.97 | 1.484 | 2.995 |
| 10 | 12774.90 | 10.270 | 17.4 | 1.466 | 3.073 |
| 11 | 13803.52 | 10.181 | 34.2 | 1.450 | 3.158 |
| 12 | 14802.51 | 10.599 | 20.4 | 1.436 | 3.238 |
| 13 | 15781.39 | 10.512 | 37.1 | 1.422 | 3.316 |

| (2)^4^Δ_5/2_ | | | | | |
| --- | --- | --- | --- | --- | --- |
| V | E_v_  (cm^-1^) | B_v_  (cm^-1^) | D_v_ ×10^4^  (cm^-1^) | R_min_  (Å) | R_max_  (Å) |
| 0 | 656.57 | 4.220 | 1.79 | 1.847 | 2.187 |
| 1 | 1935.74 | 4.133 | 1.43 | 1.758 | 2.311 |
| 2 | 3260.75 | 4.099 | 1.74 | 1.699 | 2.422 |
| 3 | 4537.12 | 8.563 | 7.31 | 1.656 | 2.518 |
| 4 | 5789.09 | 9.414 | 0.84 | 1.616 | 2.602 |
| 5 | 7024.37 | 9.071 | 8.77 | 1.582 | 2.681 |
| 6 | 8234.38 | 9.356 | 8.09 | 1.557 | 2.760 |
| 7 | 9402.93 | 10.339 | 0.59 | 1.533 | 2.841 |
| 8 | 10536.65 | 9.973 | 5.54 | 1.512 | 2.923 |
| 9 | 11630.38 | 10.308 | 3.81 | 1.493 | 3.004 |
| 10 | 12697.46 | 10.275 | 8.63 | 1.476 | 3.085 |
| 11 | 13731.74 | 10.218 | 17.9 | 1.461 | 3.165 |
| 12 | 14736.03 | 10.117 | 34.6 | 1.447 | 3.224 |
| 13 | 15714.84 | 10.542 | 21.3 | 1.434 | 3.324 |

| (2)^4^Δ_7/2_ | | | | | |
| --- | --- | --- | --- | --- | --- |
| V | E_v_  (cm^-1^) | B_v_  (cm^-1^) | D_v_ ×10^4^  (cm^-1^) | R_min_  (Å) | R_max_  (Å) |
| 0 | 595.71 | 4.175 | 1.88 | 1.852 | 2.207 |
| 1 | 1833.99 | 4.141 | 1.28 | 1.763 | 2.313 |
| 2 | 3174.91 | 4.112 | 1.91 | 1.695 | 2.424 |
| 3 | 4445.60 | 8.562 | 8.62 | 1.651 | 2.513 |
| 4 | 5697.03 | 9.418 | 1.01 | 1.611 | 2.597 |
| 5 | 6920.30 | 9.070 | 10.3 | 1.580 | 2.685 |
| 6 | 8095.27 | 9.358 | 9.09 | 1.554 | 2.769 |
| 7 | 9248.16 | 10.346 | 0.60 | 1.532 | 2.850 |
| 8 | 10379.33 | 9.980 | 5.43 | 1.512 | 2.925 |
| 9 | 11479.52 | 10.315 | 3.77 | 1.494 | 3.006 |
| 10 | 12548.50 | 10.284 | 8.60 | 1.478 | 3.089 |
| 11 | 13573.90 | 10.230 | 17.7 | 1.463 | 3.170 |
| 12 | 14578.45 | 10.135 | 33.8 | 1.449 | 3.247 |
| 13 | 15561.03 | 10.557 | 20.1 | 1.436 | 3.326 |
| 14 | 16515.93 | 10.459 | 37.4 | 1.424 | 3.405 |

| (2)^4^Π_3/2_ | | | | | |
| --- | --- | --- | --- | --- | --- |
| V | E_v_  (cm^-1^) | B_v_  (cm^-1^) | D_v_ ×10^4^  (cm^-1^) | R_min_  (Å) | R_max_  (Å) |
| 0 | 698.77 | 4.232 | 1.55 | 1.853 | 2.169 |
| 1 | 2092.05 | 4.213 | 1.53 | 1.756 | 2.290 |
| 2 | 3479.58 | 4.173 | 1.83 | 1.687 | 2.396 |
| 3 | 4793.88 | 4.048 | 1.88 | 1.644 | 2.497 |
| 4 | 6040.05 | 8.854 | 25.9 | 1.609 | 2.586 |
| 5 | 7280.46 | 10.135 | 1.19 | 1.580 | 2.676 |
| 6 | 8474.81 | 9.426 | 21.4 | 1.555 | 2.758 |
| 7 | 9650.84 | 9.734 | 19.1 | 1.532 | 2.838 |
| 8 | 10789.97 | 10.438 | 5.77 | 1.512 | 2.919 |
| 9 | 11901.58 | 9.992 | 34.9 | 1.493 | 2.995 |
| 10 | 12986.22 | 10.369 | 19.7 | 1.476 | 3.072 |
| 11 | 14040.11 | 10.251 | 56.5 | 1.4600 | 3.158 |
| 12 | 15054.66 | 10.676 | 37.0 | 1.446 | 3.235 |
| 13 | 16030.67 | 10.594 | 56.5 | 1.432 | 3.318 |
| 14 | 16973.04 | 9.831 | 75.1 | 1.420 | 3.401 |
